# Non-classical tissue monocytes and two functionally distinct populations of interstitial macrophages populate the mouse lung

**DOI:** 10.1038/s41467-019-11843-0

**Published:** 2019-09-03

**Authors:** Joey Schyns, Qiang Bai, Cecilia Ruscitti, Coraline Radermecker, Sebastiaan De Schepper, Svetoslav Chakarov, Frédéric Farnir, Dimitri Pirottin, Florent Ginhoux, Guy Boeckxstaens, Fabrice Bureau, Thomas Marichal

**Affiliations:** 1 Laboratory of Immunophysiology, GIGA Institute, Liège University, Avenue de l’Hôpital 11, B34, 4000 Liège, Belgium; 2 Laboratory of Cellular and Molecular Immunology, GIGA Institute, Liège University, Avenue de l’Hôpital 11, B34, 4000 Liège, Belgium; 3 Faculty of Veterinary Medicine, Liège University, Boulevard de Colonster 20, 4000 Liège, Belgium; 4 Translational Research Center for Gastrointestinal Disorders, Department of Chronic Diseases, Metabolism and Ageing, KU Leuven, Herestraat 49, O&N1, Box 701, 3000 Leuven, Belgium; 5 Singapore Immunology Network (SIgN), A*STAR, Biomedical Grove 8a, 138648 Singapore, Singapore; 6 FARAH Institute, Liège University, Boulevard de Colonster 20, 4000 Liège, Belgium; 7 Shangai Institute of Immunology, Shanghai JiaoTong University School of Medicine, South Chongqing Road 280, 200002 Shanghai, China; 8 WELBIO, Walloon Excellence in Life Sciences and Biotechnology, Wallonia, Belgium

**Keywords:** Monocytes and macrophages, Preclinical research

## Abstract

Resident tissue macrophages (RTM) can fulfill various tasks during development, homeostasis, inflammation and repair. In the lung, non-alveolar RTM, called interstitial macrophages (IM), importantly contribute to tissue homeostasis but remain little characterized. Here we show, using single-cell RNA-sequencing (scRNA-seq), two phenotypically distinct subpopulations of long-lived monocyte-derived IM, i.e. CD206^+^ and CD206^−^IM, as well as a discrete population of extravasating CD64^+^CD16.2^+^ monocytes. CD206^+^ IM are peribronchial self-maintaining RTM that constitutively produce high levels of chemokines and immunosuppressive cytokines. Conversely, CD206^−^IM preferentially populate the alveolar interstitium and exhibit features of antigen-presenting cells. In addition, our data support that CD64^+^CD16.2^+^ monocytes arise from intravascular Ly-6C^lo^ patrolling monocytes that enter the tissue at steady-state to become putative precursors of CD206^−^IM. This study expands our knowledge about the complexity of lung IM and reveals an ontogenic pathway for one IM subset, an important step for elaborating future macrophage-targeted therapies.

## Abstract (web summary)

Functional diversity of tissue-resident macrophages and signals governing their ontogeny and turnover remain unknown for the majority of tissues. Here the authors describe two phenotypically and functionally distinct long-lived populations of lung interstitial macrophages and their putative blood-derived monocytic precursor.

## Introduction

Resident tissue macrophages (RTM) are present in most mammalian tissues. Historically known for their roles in host defense and clearance of dead cells, RTM are now recognized as an integral part of the tissues in which they reside, where they can contribute to a wide range of physiological and pathological processes^1–3^.

RTM populations are very heterogeneous, phenotypically and functionally^1–3^, and the tissue of residence is thought to be a major driver of such diversity^4,5^. According to the niche model, RTM are imprinted by niche-derived tissue-instructive signals that trigger expression of specific differentiation programs, thus tailoring a particular identity, i.e., a phenotypic and functional specialization that fulfills the functional needs of a given tissue^5^. Supporting this, recent mouse studies have shown that distinct precursors have the potential to give rise to the same particular RTM population when the niche is empty^5–8^.

In mice, the well-known alveolar macrophages (AM) differentiate from fetal monocytes, are maintained by self-renewal and are specialized in removal and recycling of surfactant molecules^9–11^. Besides AM, non-alveolar lung macrophages, i.e., the interstitial macrophages (IM), have been shown to contribute to lung immune homeostasis by spontaneously producing the immunosuppressive cytokine IL-10 and preventing the development of aberrant type 2 allergic responses against inhaled allergens^12–15^. In addition, they may substantially contribute to the reduced risk of asthma in a microbe-rich environment (i.e., the so-called hygiene hypothesis^16,17^). Indeed, we have reported that exposure to bacterial unmethylated CpG-DNA (CpG) expands tolerogenic IM from monocyte precursors, thereby conferring robust protection against allergic asthma^18^. IL-10-producing IM have also been described in humans^19^, and clinical evidence suggests that they may be functionally impaired in asthmatic patients^20^.

Despite their functional relevance, IM were long merely investigated as a bulk population^12–14,18^. In 2017, Gibbings et al. proposed the existence of three phenotypically distinct IM populations in the steady-state lung based on the differential expression of MHC-II and CD11c^21^. More recently, Chakarov and colleagues identified two conserved monocyte-derived IM subpopulations across tissues, in mice and humans^22^. In the mouse lung, they characterized nerve-associated Lyve-1^lo^MHC-II^hi^ and blood vessel-associated Lyve-1^hi^MHC-II^lo^ monocyte-derived IM subsets, supporting that the lung IM pool is heterogeneous and encompasses distinct populations carrying their own identity.

Here, we analyze >3000 lung tissue mononuclear cells expressing the high affinity immunoglobulin gamma Fc receptor (Fcgr1, CD64) by droplet-based single-cell RNA-sequencing (scRNA-seq) in adult mice. Our study independently confirms the existence of two main subpopulations of lung IM^22^ and further expands our knowledge about their origin, half-life, localization, functional properties and dynamics upon local exposure to microbial products. Moreover, we uncover a discrete population of extravascular NR4A1-dependent monocytes transitioning from intravascular Ly-6C^lo^ patrolling monocytes towards a specific subset of IM. These results contribute to a better appreciation of the diversity of the lung mononuclear phagocyte system (MPS), an important step toward greater precision and effectiveness of macrophage-targeted therapies.

## Results

### Two subsets of IM and monocytes populate the mouse lung

**Fig. 1:**
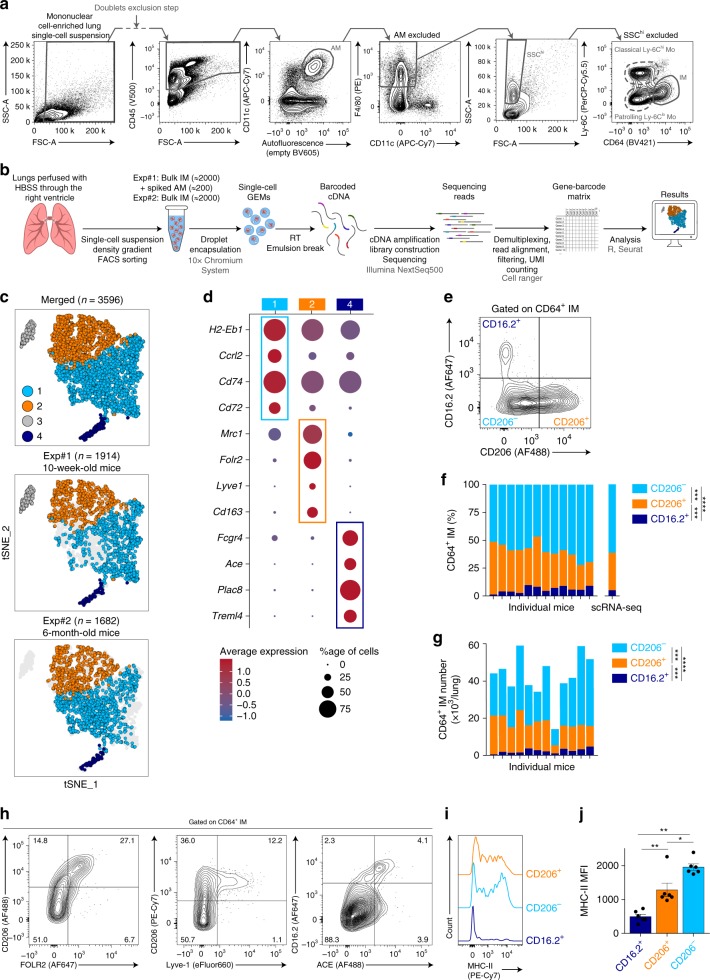
ScRNA-seq analysis of CD64^+^ mononuclear cells in lungs of naive C57BL/6 WT mice. **a** Gating strategy used for FACS sorting prior to scRNA-seq experiments. **b** Experimental pipeline of scRNA-seq experiments. **c** *t-*SNE plots depicting the CD64-expressing cells analyzed by scRNA-seq. *n* indicates the number of cells analyzed after quality control and filtering. **d** Dot plots showing average expression of the indicated genes and percentages of cells expressing the genes within each cluster. Examples of transcripts significantly differentially regulated (*P*_*adj*_ < 10^−2^) between Cluster 4, 2 or 1 vs. the 2 other clusters are depicted. **e** Representative contour plot of CD16.2 and CD206 expression within CD64^+^ IM, whose quantification is shown in (**f**). **f** Percentage of each mononuclear phagocyte subset among CD64^+^ bulk IM, assessed by flow cytometry. Stacked bars represent individual mice, and the % of cells per cluster as identified by scRNA-seq (right bar). **g** Numbers of each mononuclear phagocyte subset within the steady-state lung. Stacked bars represent individual mice. **h** Representative contour plot of the indicated markers within CD64^+^ IM. Numbers indicate the percentage of cells within the respective gates. The plots are representative of one of 6 individual mice analyzed, each of them giving similar results. **i** Representative histograms of surface MHC-II expression within each mononuclear phagocyte subset, whose quantification is shown in (**j**). **j** Quantification of MHC-II MFI. **f**, **g** Data show individual mice and are pooled from 3 independent experiments (*n* = 12). **j** Data show mean ± s.e.m. and are pooled from 2 independent experiments, each symbol representing individual mice (*n* = 6). *P* values were calculated using non-parametric **f**, **g** Friedman or **j** Mann–Whitney tests for pairwise comparisons. *P < 0.05; **P < 10^−2^; ***P < 10^−3;^ ****P < 10^−4^. Source data are provided as a Source Data file. AM, alveolar macrophage; Exp, experiment; GEM, gel bead in emulsion; IM, interstitial macrophage (sorted in bulk, as shown in **a**); MFI, Mean Fluorescence Intensity; Mo, monocyte; RT, reverse transcription

To map mouse lung tissue macrophages (i.e., lung IM) in naive C57BL/6 female wild-type (WT) mice, we performed scRNA-seq using the 10x Genomics platform^23^. Lung IM were defined as singlet mononuclear cell-enriched CD45^+^ non-autofluorescent SSC^lo^F4/80^+^CD11c^−^Ly-6C^lo^CD64^+^ cells^18^ (Fig. 1a, and Supplementary Table 1). Exclusion of lung-resident F4/80^+^Siglec-F^+^ eosinophils^24^ based on high SSC was efficient and resulted in minimal loss of IM (Supplementary Fig. 1). In a first experiment, 10-week-old mice were used and a total of 1715 IM, together with 199 AM, were analyzed (Fig. 1b, and Supplementary Fig. 2). In addition, a second scRNA-seq experiment was performed through an independent platform using older mice (i.e., 6-month-old) coming from a different animal facility, and 1682 IM were analyzed (Fig. 1b, and Supplementary Fig. 2).

Non-linear dimensional reduction (*t*-distributed stochastic neighbor embedding [*t*-SNE]) and graph-based clustering of single cells merged from both experiments identified 4 transcriptionally distinct clusters of monocytes/macrophages (Fig. 1c, and Supplementary Fig. 3a, b)^25,26^. Cluster 3 represented AM (Supplementary Fig. 3c, d)^11^, and Clusters 1, 2, and 4 were distributed in the same proportions in both experiments and were characterized by higher expression of *Cx3cr1, Mafb, Cd14*, and *Cd74* as compared to AM (Supplementary Fig. 3c, d), supporting the contention that it comprised lung tissue IM.

Clusters 1, 2, and 4 exhibited unique transcriptional signatures (Supplementary Fig. 4a, b), including upregulation of transcripts encoding proteins detectable by flow cytometry: MHC-II-related transcripts (e.g., *H2-Eb1*, *H2-Ab1*, *Cd74*) in Cluster 1; transcripts encoding macrophage mannose receptor (*Mrc1*, encoding CD206), the scavenger receptor *Cd163*, folate receptor beta precursor (*Folr2*) and lymphatic endothelium hyaluronan receptor-1 (*Lyve1*) in Cluster 2; and transcripts encoding angiotensin-converting enzyme (*Ace*) and low affinity immunoglobulin gamma Fc region receptor IV (*Fcgr4*, encoding CD16.2) in Cluster 4 (Fig. 1d, and Supplementary Fig. 4c).

Using antibodies directed against CD206 and CD16.2, we showed that expression of these markers was mutually exclusive within CD64^+^ IM and 3 subpopulations were identified: a minor population of CD16.2^+^CD206^−^ cells, which co-expressed ACE and corresponded to Cluster 4 (dark blue cells, defined as [CD64^+^]CD16.2^+^ [monocytes] hereafter, Fig. 1e–h); CD16.2^−^CD206^+^ cells, in which a fraction uniquely expressed Lyve-1 and FOLR2, and corresponding to Cluster 2 (orange cells, defined as CD206^+^ [IM] hereafter, Fig. 1e–h); and CD16.2^−^CD206^−^ cells, which were expressing significantly higher levels of MHC-II as compared to the other subsets and corresponded to Cluster 1 (light blue cells, defined as CD206^−^[IM] hereafter, Fig. 1e–j). Of note, CD206^−^and CD206^+^ subsets largely overlapped with Lyve-1^lo^MHC-II^hi^ and Lyve-1^hi^MHC-II^lo^ IM subsets described by Chakarov et al.^22^, as well as with IM3 and IM1/IM2 subsets described by Gibbings et al., respectively (Supplementary Fig. 5).

**Fig. 2:**
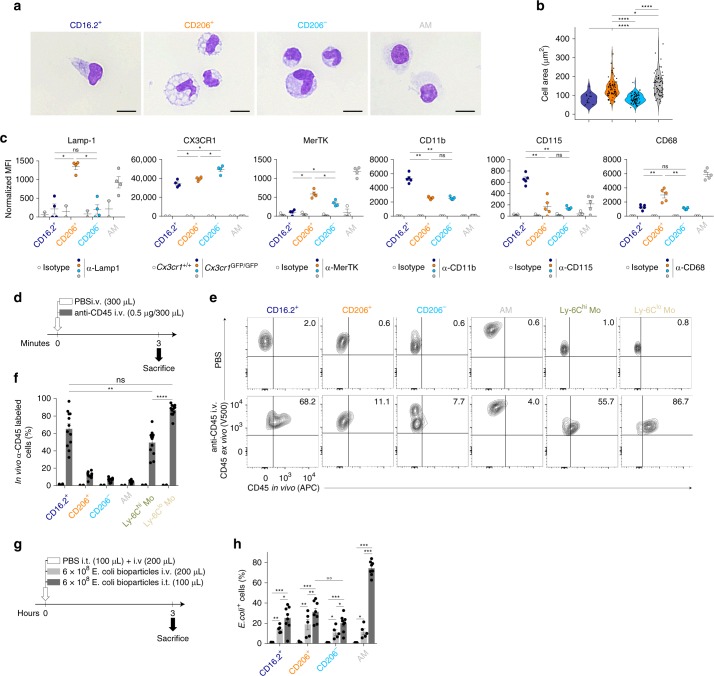
Morphology, phenotype, tissue association and phagocytic abilities of lung CD64^+^ subpopulations. **a** Representative photograph of the indicated FACS-sorted populations. **b** Quantification of the size of cells presented as violin plots (height: cell area; width abundance of cells) and individual dots representing single cells. **c** Quantification of expression of the indicated markers as compared to control cells. **d** Experimental outline for panels (**e**, **f**). **e** Representative contour plots showing binding of anti-CD45 in vivo vs. anti-CD45 ex vivo antibodies on the indicated cell populations. Numbers indicate the percentage of double-positive cells. **f** Percentage of cells positive for anti-CD45 in vivo and ex vivo stainings within the indicated populations. **g** Experimental outline for panel (**h**). **h** Percentage of *E. coli* bioparticle-positive cells 3 h after i.v. or i.t. administration. Data show (**b**) individual cells pooled from 3 independent sorting experiments (CD16.2^+^, CD206^+^, CD206^−^, AM: *n* = 16,77,75,72, respectively), or (**c, f, h**) mean ± s.e.m., as well as individual mice (**c**, *n* = 4-5; **f**, *n* = 12; **h**, *n* = 5–8/group), and are pooled from 2 independent experiments. *P-*values were calculated using (**c**, **f**) non-parametric Mann–Whitney tests for pairwise comparisons or (**h**) a linear mixed model on log(y+1)-transformed values with Tukey’s post hoc test. *P < 0.05; **/°°*P* < 10^−2^; ***P < 10^−3^; ****P < 10^−4^; ns, not significant. Empty circles compare % of *E. coli*^+^ cells after i.t. injection in CD206^+^ vs. CD206^−^ IM subsets. Source data are provided as a Source Data file. i.t., intratracheal; i.v., intravenous; MFI, Mean Fluorescence Intensity. Scale bar = 10 µm

Morphologically, CD206^+^ IM uniquely displayed vacuoles in their cytoplasm and a larger size as compared to CD206^−^ IM and CD64^+^CD16.2^+^ cells (Fig. 2a, b). Immunostaining against Lamp-1, a lysosomal marker, suggested that the vacuoles seen in CD206^+^ IM were lysosomes (Fig. 2c, and Supplementary Fig. 6). Phenotypically, CD206^−^IM expressed higher levels of CX3CR1, whereas CD206^+^ IM expressed higher levels of the macrophage-associated markers MerTK and CD68 as compared to the 2 other subpopulations (Fig. 2c, and Supplementary Fig. 6). Moreover, CD64^+^CD16.2^+^ cells expressed higher levels of CD11b and CD115 and lower levels of MerTK as compared to both IM subsets (Fig. 2c, and Supplementary Fig. 6), consistent with the idea that CD64^+^CD16.2^+^ cells were monocytes.

Next, we injected fluorescent-conjugated anti-CD45 antibodies intravenously (i.v.) to label intravascular leukocytes before the sacrifice (Fig. 2d). AM, CD206^+^, and CD206^−^ IM were only marginally stained by such antibodies (Fig. 2e, f), confirming that these cells were mainly extravascular. Expectedly, nearly all patrolling Ly-6C^lo^ monocytes and a majority of classical Ly-6C^hi^ monocytes were labeled, confirming the existence of tissue-associated Ly-6C^hi^ monocytes (Fig. 2e, f)^18,27^. However, the percentage of CD64^+^CD16.2^+^ monocytes that were stained with the anti-CD45 in vivo exhibited a high variability and was significantly lower than the one of patrolling Ly-6C^lo^ monocytes (Fig. 2e, f), suggesting that a substantial fraction of CD64^+^CD16.2^+^ monocytes was truly located in the lung tissue.

We also sought to test the ability of each subpopulation to engulf large particles (i.e., *E. coli* bioparticles conjugated with a pH-sensitive dye), i.e. a functional hallmark of macrophages (Fig. 2g). Like AM, CD64^+^CD16.2^+^ monocytes, CD206^+^ and CD206^−^ IM were able to phagocyte airborne and blood-borne particles, with significantly higher percentages of cells when particles were injected i.t. as compared to i.v. (Fig. 2h). After i.t. injection, percentages of fluorescent CD206^+^ IM were significantly higher than those of CD206^−^ IM, which might indicate a preferential localization around the airways (Fig. 2h).

So far, our data suggest that, in addition to dendritic cells (DCs) and tissue Ly-6C^hi^ classical monocytes^18,27^, the lung MPS comprises 3 subpopulations of Ly-6C^lo^CD64^+^ mononuclear phagocytes, namely CD206^+^ IM, CD206^−^ IM, and non-classical CD64^+^CD16.2^+^ monocytes.

### IM subsets are long-lived, unlike NR4A1-dependent monocytes

**Fig. 3:**
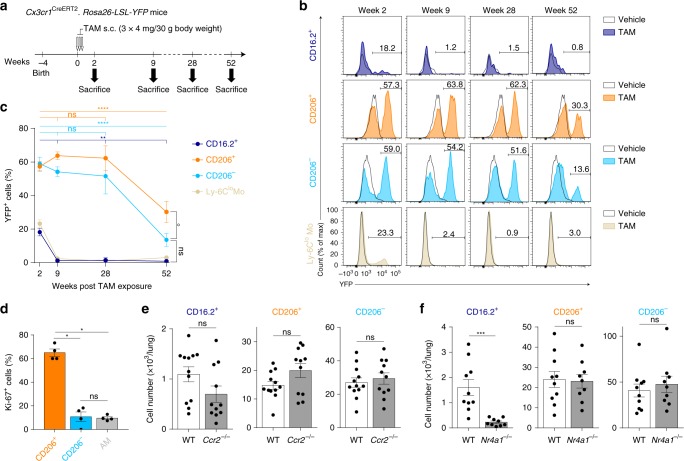
Maintenance of lung tissue CD64^+^ mononuclear phagocytes in adult C57BL/6 mice. **a** Experimental outline for panels (**b**, **c**). Briefly, at 4 weeks of age, *Cx3cr1*^CreERT2^*.Rosa26-LSL-YFP* mice were treated with TAM s.c. 3 times, 48h apart. Mice were analyzed for YFP expression 2, 9, 28, and 52 weeks later. **b** Representative histograms of YFP expression within the indicated populations. Numbers indicate the percentage of YFP^+^ cells, as quantified in (**c**). **c** Percentage of YFP^+^ cells within the indicated populations, assessed by flow cytometry. **d** Percentages of Ki-67^+^ cells in the indicated populations. **e**, **f** Absolute numbers of the indicated cell populations in the lungs of **e** *Ccr2*^*−/−*^ or **f** *Nr4a1*^*−/−*^ and control WT mice. **c–f** Data show mean ± s.e.m., as well as individual mice in (**d–f**) (**c**, *n* = 10; **d**, *n* = 4; **e**, **f**, *n* = 9–12) and are pooled from 2 to 3 independent experiments. *P*-values were calculated using (**c**) a two-way ANOVA with Tukey’s post hoc test, **d** non-parametric Mann–Whitney test for pairwise comparisons or **e**, **f** a two-tailed unpaired Student’s *t*-test. °/*P < 0.05; **P < 10^−2^; ***P < 10^−3^; ****P < 10^−4^; ns, not significant. In **c** the empty circle compares CD206^+^ and CD206^−^IM at week 52. Source data are provided as a Source Data file. s.c., subcutaneous; TAM, tamoxifen

While previous studies have provided evidence that IM were monocyte-derived cells in adults^18,21,22,28^, they did not exclude the possibility that part of the IM compartment may be self-maintaining in the tissue. To assess the half-life of IM subpopulations, we used the tamoxifen(TAM)-inducible *Cx3cr1*^CreERT2^*.Rosa26-LSL-YFP* fate-mapping mouse model^29^, and TAM-injected *Cx3cr1*^CreERT2^*.Rosa26-LSL-YFP* mice were longitudinally evaluated for yellow fluorescent protein (YFP) labeling in lung mononuclear phagocytes (Fig. 3a). Two weeks after injection, YFP^+^ cells were uniquely found among CD64^+^ subpopulations and Ly-6C^lo^ patrolling monocytes, while YFP was virtually absent in lung Ly-6C^hi^ classical monocytes or DCs (Fig. 3b, c, and Supplementary Fig. 7). Of note, the majority of CD206^+^ and CD206^−^ IM subpopulations were YFP^+^, whereas less than 20% of the CD64^+^CD16.2^+^ subset was YFP^+^, similarly to what was observed in Ly-6C^lo^ patrolling monocytes (Fig. 3b, c). In addition, CD64^+^CD16.2^+^ cells were all replaced by YFP^−^ monocytes at week 9 (Fig. 3b, c). Nine and 28 weeks after TAM treatment, the percentages of YFP^+^CD206^+^ and YFP^+^CD206^−^ IM remained high and were not significantly different from those observed 2 weeks post-injection (Fig. 3b, c), supporting that both IM subsets could self-maintain in adults. However, percentages of YFP^+^CD206^+^ and YFP^+^CD206^−^ cells were significantly decreased at week 52 as compared to week 2, confirming that both subpopulations were slowly replaced by YFP^−^ monocytes over time (Fig. 3b, c). Interestingly, more than half of the YFP^+^ labeling present at week 2 was still detected 50 weeks later in CD206^+^ IM, as opposed to less than 24% in CD206^−^IM (Fig. 3b, c). In addition, levels of the proliferation marker Ki-67 were significantly greater in CD206^+^ IM as compared to CD206^−^ IM and AM (Fig. 3d), suggesting that CD206^+^ IM could proliferate and had an increased self-maintenance potential as compared to CD206^−^IM.

We have previously shown that bulk IM numbers were not significantly affected in 6–10-week-old *Ccr2*^*−/−*^ or *Nr4a1*^*−/−*^ mice^18^, whose numbers of blood Ly-6C^hi^ and Ly-6C^lo^ monocytes are impaired, respectively^30,31^ (Supplementary Fig. 8). While numbers of CD206^+^ and CD206^−^ IM were similar in WT, *Ccr2*^*−/−*^ and *Nr4a1*^*−/−*^ mice (Fig. 3e, f), numbers of CD64^+^CD16.2^+^ monocytes were significantly reduced in *Nr4a1*^*−/−*^ mice as compared to WT mice (Fig. 3e, f), like those of Ly-6C^lo^ monocytes, demonstrating that CD64^+^CD16.2^+^ monocytes depended on NR4A1 for their presence in the lung.

The similarities between CD64^+^CD16.2^+^ monocytes and intravascular Ly-6C^lo^ patrolling monocytes (i.e., half-life, dependence on NR4A1 and surface phenotype [Supplementary Fig. 9a, b]) supported the possibility that CD64^+^CD16.2^+^ monocytes actually derived from Ly-6C^lo^ patrolling monocytes, but expressed CD64 and were partly extravascular. If CD64^+^CD16.2^+^ monocytes enter the tissue, they should be imprinted by tissue-instructive signals and, hence, exhibit a transcriptomic profile that is distinct from the one of intravascular Ly-6C^lo^ monocytes. Hence, we compared CD64^−^Ly-6C^lo^ patrolling monocytes (Supplementary Fig. 9c–e) with CD64^+^CD16.2^+^ monocytes by scRNA-seq and found that they segregated in separated clusters (Supplementary Fig. 9f). Moreover, we found that many of the most upregulated transcripts in CD64^+^CD16.2^+^ monocytes (Supplementary Fig. 9g–i) were also found to be significantly upregulated in IM as compared to AM (see Supplementary Fig. 3d). These data support the notion that CD64^+^CD16.2^+^ monocytes can be distinguished from Ly-6C^lo^ patrolling monocytes by their expression of tissue-specific IM-related genes, likely as a result of tissue-derived imprinting.

Altogether, our data identified two main subsets of long-lived monocyte-derived IM, with CD206^+^ IM exhibiting a greater half-life than CD206^− ^IM, as well as short-lived NR4A1-dependent CD64^+^CD16.2^+^ monocytes.

### CD206^+^ and CD206^−^IM preferentially populate distinct niches

**Fig. 4:**
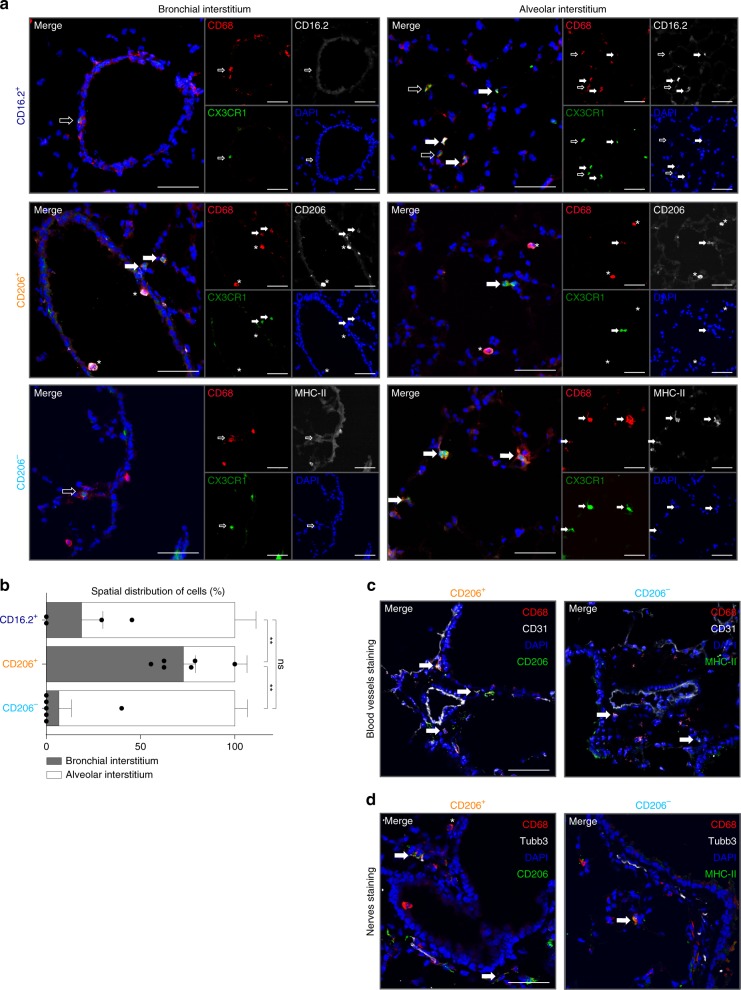
Localization of lung CD64^+^CD16.2^+^ monocytes, CD206^+^ IM and CD206^−^ IM **a** Confocal microscopy pictures of lung sections from *Cx3cr1*^GFP/GFP^ mice (CX3CR1 [green]; CD68 [red]; DAPI [blue]; CD16.2, MHC-II or CD206 [white]). CD64^+^CD16.2^+^ monocytes, CD206^+^ IM and CD206^−^ IM were identified as CX3CR1^+^CD68^+^CD16.2^+^, CX3CR1^+^CD68^+^CD206^+^ and CX3CR1^+^CD68^+^MHC-II^+^cells, respectively. Asterisks indicate CX3CR1^−^CD68^+^ AM; plain or empty arrows indicate CX3CR1^+^CD68^+^ cells expressing or not the marker of interest (i.e., CD16.2, CD206 or MHC-II), respectively. **b** Preferential distribution of the indicated populations in the peribronchial/perivascular area *vs*. the alveolar parenchyma. **c**, **d** Lung sections of C57BL/6 WT mice were analyzed: CD68 [red]; **c** CD31 or **d** Tubb3 [white]; DAPI [blue]; MHC-II, CD206 or CD16.2 [green]). CD206^+^ IM, CD206^− ^IM and CD64^+^CD16.2^+^ monocytes were identified as CD68^+^CD206^+^, CD68^+^MHC-II^+^ and CD68^+^CD16.2^+^ cells, respectively. **b** Data show mean ± s.e.m. and are pooled from 2 independent batches of mice (*n* = 4–6). *P-*values were calculated using a Kruskal–Wallis test, and pairwise comparisons were estimated using Mann–Whitney tests. **P < 10^−2^; ns, not significant. Source data are provided as a Source Data file. Scale bars = (**a)** 100 µm; (**c,d**) 50 µm

To assess the preferential localization of the two IM subpopulations and of CD64^+^CD16.2^+^ monocytes, we used confocal microscopy. Lung sections of *Cx3cr1*^GFP/GFP^ mice were stained with antibodies directed against GFP, CD68 (as a macrophage marker) and either CD206, MHC-II or CD16.2. Of note, we observed only a minor fraction of CD68^+^ cells expressing simultaneously CD206 and MHC-II using a combined anti-CD206 and anti-MHC-II staining (Supplementary Fig. 10). Hence, CX3CR1^+^CD68^+^CD206^+^, CX3CR1^+^CD68^+^MHC-II^+^ and CX3CR1^+^CD68^+^CD16.2^+^ triple-positive cells were quantified in multiple sections and fields to evaluate the spatial distribution of CD206^+^ IM, CD206^−^ IM and CD64^+^CD16.2^+^ monocytes, respectively (Fig. 4a, b). On the one hand, we found that CD206^+^ IM were preferentially found in the bronchial interstitium, whereas CD206^−^ IM and CD64^+^CD16.2^+^ monocytes were mainly located in the alveolar interstitium (Fig. 4a, b). On the other hand, since Chakarov et al. reported that peribronchial Lyve-1^lo^MHC-II^hi^ and Lyve-1^hi^MHC-II^lo^ IM subsets were mainly associated with nerves and blood vessels, respectively^22^, we used antibodies against CD31 and Tubb3 to stain nerves and endothelial cells. While CD206^+^ IM were associated with blood vessels (Fig. 4c), the preferential localization of peribronchial CD206^−^ IM next to nerves was, however, less obvious, which is likely due to the close association of blood vessels and nerves in the peribronchial areas of the lung (Fig. 4d).

These data support that the two IM subpopulations are found in distinct micro-anatomical niches, which may dictate specific functional specializations.

### CD206^+^ and CD206^−^IM exhibit distinct functional properties

Next, we performed Gene Ontology (GO) enrichment analyses to gain insights into the functional properties of CD64^+^CD16.2^+^ monocytes and IM subpopulations. First, comparison between CD64^+^CD16.2^+^ monocytes and IM subsets revealed an enrichment, in CD64^+^CD16.2^+^ monocytes, in transcripts involved in leukocyte cell–cell adhesion, integrin-mediated signaling pathway, positive regulation of cytoskeleton organization and myeloid leukocyte migration (Table 1, Supplementary Fig. 11 and Supplementary Table 2), supporting the possibility that CD64^+^CD16.2^+^ monocytes may be actively extravasating in the lung tissue. Second, we found that the upregulated transcripts in CD206^+^ IM were enriched for processes related to the positive regulation of leukocyte chemotaxis, response to wounding and receptor-mediated endocytosis, consistent with their phenotype and lysosomal vacuoles (Table 1, Supplementary Fig. 11 and Supplementary Table 2). Third, CD206^−^ IM had increased expression of transcripts associated with antigen processing and presentation, regulation of T cell activation and defense response (Table 1, Supplementary Fig. 11 and Supplementary Table 2).

**Table 1:** Gene Ontology analysis of the transcriptomic profiles of IM subpopulations

Cluster	Biological process	Reference	Expected	Specific genes	Fold enrichment	*P*-value
1 (CD206^−^)	Defense response	1296	7.09	33	4.65	7.62 × 10^−10^
	Regulation of T cell activation	297	1.63	14	8.61	1.40 × 10^−5^
	Antigen processing and presentation	95	0.52	9	17.31	4.76 × 10^−5^
	Cellular response to lipopolysaccharide	197	1.08	11	10.20	1.65 × 10^−4^
2 (CD206^+^)	Receptor-mediated endocytosis	130	1.07	12	11.25	1.76 × 10^−5^
	Response to wounding	331	2.72	15	5.52	1.46 × 10^−3^
	Positive regulation of leukocyte chemotaxis	85	0.70	8	11.47	8.04 × 10^−3^
	Cellular response to lipopolysaccharide	197	1.62	11	6.80	9.98 × 10^−3^
4 (CD16.2^+^)	Leukocyte cell–cell adhesion	48	1.02	12	28.22	2.81 × 10^−5^
	Integrin-mediated signaling pathway	73	1.55	13	8.38	2.20 × 10^−4^
	Positive regulation of cytoskeleton organization	206	4.38	19	4.34	2.41 × 10^−3^
	Myeloid leukocyte migration	117	2.49	13	5.23	2.88 × 10^−2^

Reference indicates the number of genes in the gene set, Expected the average number of genes expected to be present if there is no enrichment, and Specifc genes the number of genes from the gene set that are upregulated in the indicated Cluster. *P*-values were calculated using a two-tailed Mann–Whitney *U* test with Benjamini–Hochberg False Discovery Rate correction

**Fig. 5:**
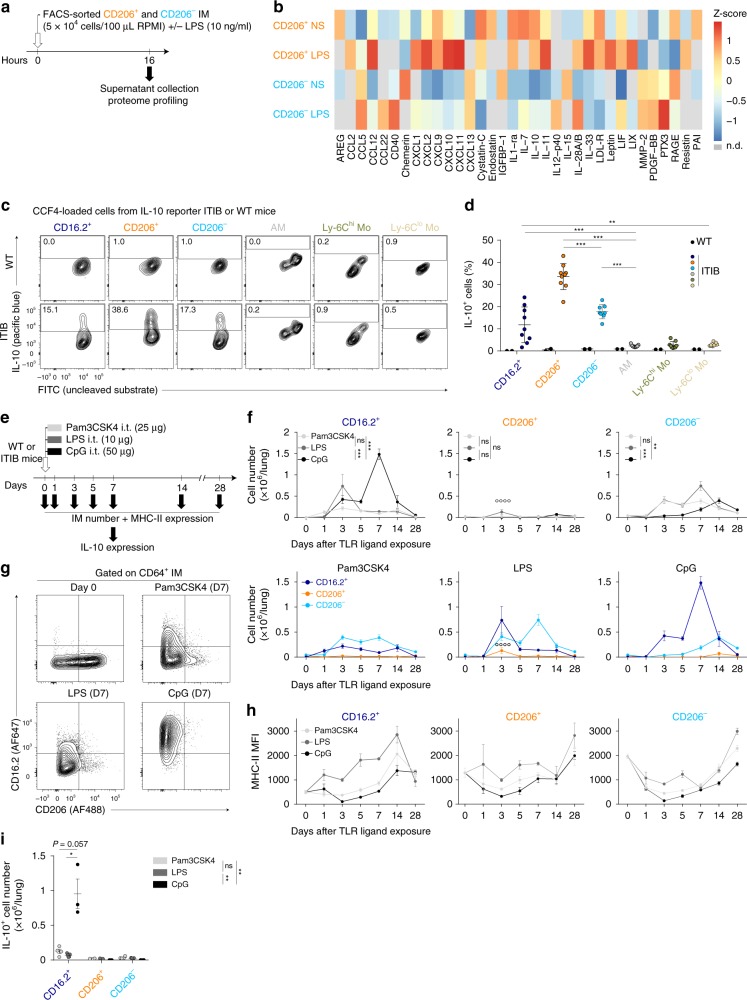
Functional properties of IM subpopulations at steady-state and dynamic regulation after airway exposure to microbial products. **a** Experimental outline for (**b**). FACS-sorted IM subpopulations were cultured ex vivo overnight with or without LPS, and supernatants were subjected to proteome profiling. **b** Heatmap depicting the relative abundance of the indicated molecules in the supernatants of non-stimulated (NS) or LPS-stimulated (LPS) lung IM subpopulations. Data represent the mean and are representative of one of 2 independent sorting experiments. **c** Representative contour plots showing steady-state IL-10 expression as assessed by detection of 450 nm fluorescence (blue fluorescent product of the cleaved CCF4 substrate) in CCF4-loaded cells isolated from IL-10-β-lactamase reporter ITIB or WT control mice. Numbers indicate % of IL-10^+^ cells within the cell populations, as quantified in (**d**). **d** Percentages of IL-10^+^ cells in the indicated populations. **e** Experimental outline for panels (**f–i**). **f** Kinetic analysis of numbers of each mononuclear phagocyte subset after i.t. instillation of Pam3CSK4, LPS, and CpG. **g** Representative contour plot of CD16.2 and CD206 expression within CD64^+^ IM from control (Day 0), and Pam3CSK4-, LPS- and CpG-injected mice 7 days after treatment. **h** Kinetic analysis of MHC-II expression within the indicated populations after Pam3CSK4, LPS or CpG treatment. **i** Numbers of IL-10^+^ cells within the indicated subpopulations 7 days after treatment. **d**, **f**, **h**, **i** Data show mean ± s.e.m., as well as individual mice in (**d, i**), and are pooled from 2 independent experiments (**d**, *n* = 9; **f**, **h**, *n* = 5–6/time point; **i**, *n* = 3–5). *P-*values were calculated using **d**, **i** non-parametric Mann–Whitney tests for pairwise comparisons or **f**, **h** two-way ANOVA with Tukey’s post hoc tests. *P < 0.05; **P < 10^−2^; ***P < 10^−3^; ****/°°°°*P* < 10^−4^; ns, not significant. In **f** the empty circles compare numbers of CD206^+^ IM 3 days after LPS vs. day 0. Source data are provided as a Source Data file. i.t., intratracheal

To complement these mRNA data at the protein level, we performed a proteome profiling on the supernatants of FACS-sorted CD206^+^ and CD206^−^ IM (Fig. 5a, b). CD206^+^ IM were characterized by an elevated chemokine secretory profile (e.g., CXCL11, CXCL10, CXCL9, CXCL2, CCL12), a higher secretion of immunoregulatory cytokines (IL-10, IL1-Ra) and factors regulating cell growth and differentiation, such as leukemia inhibitory factor (LIF), amphiregulin (AREG), or IL-7 (Fig. 5a, b). Conversely, CD206^−^ IM secreted larger amounts of Pentraxin 3 (PTX3), secreted in response to inflammatory signals and facilitating pathogen recognition^32^, the p40 subunit of the type 1 helper T cell (Th1)-differentiating cytokine IL-12, and the B and T lymphocyte chemoattractant CXCL13 and CCL5, respectively (Fig. 5a, b). GO analysis also showed that both IM subsets expressed high levels of genes implicated in the cellular response to LPS as compared to CD64^+^CD16.2^+^ monocytes (Table 1, Supplementary Fig. 11 and Supplementary Table 2). Of note, ex vivo LPS stimulation potentiated the secretion of chemokines and immunoregulatory cytokines in CD206^+^ IM, and of PTX3, IL12p40 and CCL5 in CD206^−^ IM (Fig. 5a, b).

At steady-state, we and others have shown that, upon engagement of Toll like receptor (TLR)4 (i.e., the main receptor for LPS) and the adaptor molecule MYD88, bulk IM could fulfill important tolerogenic tasks by inhibiting DC functions via IL-10-dependent mechanisms, thus preventing the development of asthma in animal models^12,18^. Proteome profiler data supported that CD206^+^ IM were the main IL-10-secreting cells (Fig. 5a, b). To validate these findings, we assessed IL-10 expression in lung monocyte/macrophage populations from IL-10-β-lactamase reporter ITIB mice^33^ (Fig. 5c, d). First, we observed that AM, Ly-6C^hi^ classical and Ly-6C^lo^ patrolling monocytes exhibited low percentages of IL-10^+^ cells (Fig. 5c, d). Second, we found that the percentage of CD206^+^ IM expressing IL-10 was significantly higher than the one of CD206^−^ IM (Fig. 5c, d). Third, we showed that the percentage of IL10^+^CD64^+^CD16.2^+^ monocytes was significantly higher than the one of IL-10^+^ patrolling Ly-6C^lo^ monocytes (Fig. 5c, d), pointing out another notable difference between CD64^+^CD16.2^+^ and patrolling monocytes.

Altogether, our data support that, in addition to their distinct phenotype and localization, IM subpopulations are characterized by unique functional properties. CD206^+^ IM exhibit a prominent tolerogenic and chemokine secretory profile, whereas CD206^−^ IM have a typical antigen-presenting cell profile. Besides IM subsets, a fraction of CD64^+^CD16.2^+^ monocytes also express IL-10, a functional hallmark of lung IM^12,15,18,19^.

### Exposure to TLR ligands differentially modulates IM subsets

We reported previously that local instillation of TLR ligands, such as Pam3CSK4, LPS, and CpG (i.e., TLR1/2, TLR4, and TLR9 ligands, respectively) promoted an expansion of bulk IM^18^. Here, we exposed mice to Pam3CSK4, LPS, and CpG and performed time-course analysis of IM subsets (Fig. 5e). Pam3CSK4 and LPS induced similar dynamic changes, characterized by transient increases in numbers of CD64^+^CD16.2^+^ cells followed by a subsequent expansion of CD206^−^ IM (Fig. 5f, g). LPS also significantly increased numbers of CD206^+^ IM at day 3 as compared to baseline (Fig. 5f). After CpG treatment, the profile was drastically different as compared to LPS or Pam3CSK4, with a more robust and sustained increase in numbers of CD64^+^CD16.2^+^ cells, reaching a peak at day 7, and a gradual increase in numbers of CD206^− ^IM peaking at day 14 (Fig. 5f, g). Of note, increases in numbers of CD206^−^ cells were associated with a drop in global MHC-II expression by those cells (days 3–7, Fig. 5h). Conversely, MHC-II expression of CD64^+^CD16.2^+^ cells gradually increased from day 5 to day 14, regardless of the treatment (Fig. 5h). Twenty-eight days after treatment, MHC-II expression was equal or even higher than day 0 in each subset, regardless of the treatment (Fig. 5h). Functionally, CpG was by far the most potent stimulus in triggering IL-10^+^ IM, which was restricted to the CD64^+^CD16.2^+^ compartment (Fig. 5i).

### RNA velocity identifies local precursors of CD206^−^IM

**Fig. 6:**
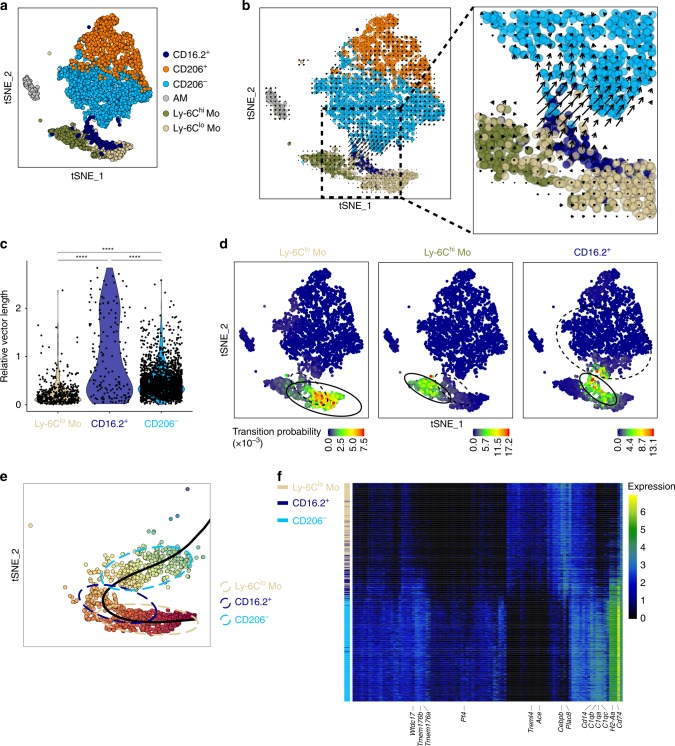
RNA velocity and trajectory analyses of lung monocyte and IM subpopulations in steady-state C57BL/6 mice. **a** *t-*SNE plot depicting the merged scRNA-seq data of lung CD64-expressing cells (see Fig. 1c), Ly-6C^lo^ patrolling monocytes (see Supplementary Fig. 9) and Ly-6C^hi^ classical monocytes (see Supplementary Fig. 12). **b** Prevalent patterns of RNA velocities substantiated by arrows and visualized on the same *t-*SNE plot as shown in (**a**). Right panel shows a higher magnification of the area depicted by a black dashed line in the left panel. (see single cell velocities in Supplementary Fig. 13). **c** Violin plot showing quantification of single cell relative 2D velocities in the indicated cell (sub)populations, as presented in Supplementary Fig. 13. **d** Visualization of single-step transition probabilities from Ly-6C^lo^ patrolling monocytes (left), Ly-6C^hi^ classical monocytes (middle) or CD64^+^CD16.2^+^ monocytes (right) to neighboring cells. Ellipses represent 95% confidence. **e**, **f** Slingshot analysis of Ly-6C^lo^ patrolling monocytes, CD64^+^CD16.2^+^ monocytes, and neighboring CD206^−^ IM. **e** Suggested pseudo-time trajectory from Ly-6C^lo^ patrolling monocytes to CD206^−^ IM. Ellipses represent 80% confidence. **f** Heatmap depicting gene expression profiles of Ly-6C^lo^ patrolling monocytes, CD64^+^CD16.2^+^ monocytes, and neighboring CD206^−^ IM ordered according to Slingshot pseudo-time trajectory. Left color bars indicate annotation by cell type

To gain insights into cell fate decisions, we applied RNA velocity analysis^34^ to our scRNA-seq datasets, i.e. IM subsets, AM, CD64^+^CD16.2^+^ monocytes, Ly-6C^lo^ patrolling and Ly-6C^hi^ classical monocytes (Supplementary Fig. 12). RNA velocity utilizes the balance between unspliced and spliced mRNAs to estimate the transition probability of individual cells^34^. Velocities, substantiated by arrows, can easily be projected on the *t*-SNE plot representing the merged scRNA-seq datasets on the basis of the similarity between the extrapolated state of a single cell and the static state of other cells in the local neighborhood (Fig. 6a, b, and Supplementary Fig. 13). Confirming the idea that both IM subsets arise from independent lineages^22^, RNA velocities of CD206^−^ and CD206^+^ IM suggested that both IM subpopulations were relatively stable and independent from each other, as no clear transition could be observed from one subpopulation to the other (Fig. 6b, and Supplementary Fig. 14).

Interestingly, RNA velocities of CD64^+^CD16.2^+^ monocytes were significantly higher than those of Ly-6C^lo^ patrolling monocytes or CD206^−^ IM, supporting their dynamic transition state, and were pointing towards CD206^−^ IM (Fig. 6b, c). Moreover, transition probability analysis of Ly-6C^lo^ patrolling and CD64^+^CD16.2^+^ monocytes suggested that they could give rise to CD64^+^CD16.2^+^ monocytes and CD206^−^ IM, respectively (Fig. 6d), supporting that CD64^+^CD16.2^+^ monocytes are mobilizable, on a timescale of hours^34^, to become CD206^−^ IM. Using Slingshot package for pseudo-time inference analysis^35^, we found a continuum from Ly-6C^lo^ monocytes towards CD206^−^ IM, with CD64^+^CD16.2^+^ monocytes as an intermediate state (Fig. 6e). Among the gene expression changes driving such transition, we observed a downregulation of a patrolling monocyte signature (e.g., *Cebpb*, encoding C/EBPβ, essential for Ly-6C^lo^ monocyte survival^36^, *Plac8*, *Treml4, Ace* [see Supplementary Fig. 9g, h]) concomitantly to an upregulation of many previously identified IM-related transcripts, including MHC-II-related transcripts (see Supplementary Figs. 3d and 9h, i) (Fig. 6f).

### NR4A1-dependent monocytes can differentiate into CD206^−^ IM

**Fig. 7:**
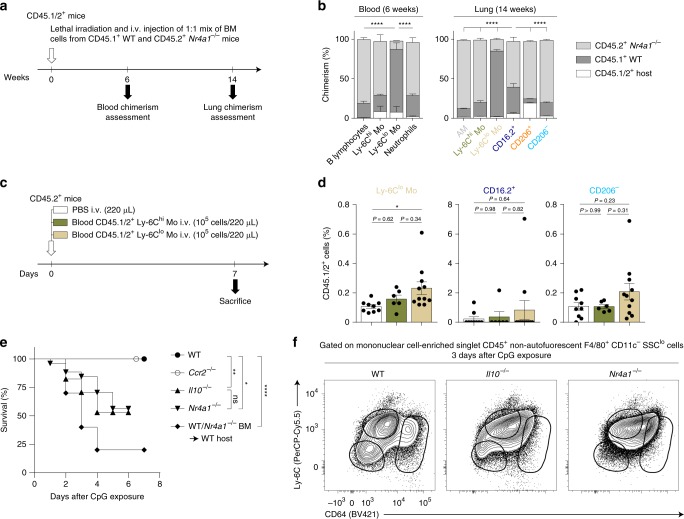
Patrolling monocyte-derived NR4A1-dependent CD16.2^+^ monocytes are local precursors of CD206^−^ IM. **a** Experimental outline for experiments using mixed BM competitive chimeras shown in (**b**). **b** Percentage of BM chimerism and radio-resistance of the indicated populations. **c** Experimental outline for (**d**). **d** Percentages of CD45.1/2^+^ cells within the indicated cell populations. **e** Survival (% of live animals) were monitored at the indicated mice after CpG treatment. *P-*values are versus CpG-injected WT mice. **f** Representative contour plot of Ly-6C and CD64, gated on singlet mononuclear cell-enriched CD45^+^ non-autofluorescent SSC^lo^F4/80^+^CD11c^−^ cells (see Fig. 1a). Data are representative of 1 of 4 mice analyzed, each giving similar patterns. Data in **b**, **d** shown mean ± s.e.m., as well as individual mice in (**d**), and are pooled from (**b**) 2 or (**d**) 3 independent experiments (**b**, *n* = 3 [blood] or 7 [lung]; **d**, *n* = 6–11/group). **e** Data were pooled from 2–4 independent experiments (*n* = 10–26/group). *P-*values were calculated using **b** a one-way ANOVA followed by Tukey’s post hoc tests, **d** non-parametric Mann–Whitney tests for pairwise comparisons or **e** Mantel–Cox tests. *P < 0.05; **P < 10^−2^; ****P < 10^−4^; ns, not significant. i.v., intravenous

Finally, we sought to validate our computational-based conclusions in vivo. First, we generated bone marrow (BM) competitive CD45.1/2 chimeras engrafted with CD45.1^+^ WT and CD45.2^+^ *Nr4a1*^*−/−*^ BM cells (Fig. 7a). Six weeks after reconstitution, >85% of blood Ly-6C^lo^ patrolling monocytes were of CD45.1^+^ WT origin, whereas less than 25% of NR4A1-independent B lymphocytes and neutrophils were of CD45.1^+^ WT origin (Fig. 7b). After 14 weeks, repopulated lung AM and both IM subsets displayed a chimerism similar to NR4A1-independent cells, confirming that such niches were repopulated by CCR2-dependent classical monocytes after lethal irradiation (Fig. 7b)^18^. Nevertheless, there was a significant enrichment, in repopulated CD64^+^CD16.2^+^ monocytes, in cells of CD45.1^+^ WT origin as compared to NR4A1-independent cells (Fig. 7b), supporting the idea that even in such extreme conditions, Ly-6C^lo^ patrolling monocytes substantially contributed to the pool of CD64^+^CD16.2^+^ monocytes. Second, we performed i.v. adoptive transfers of blood donor CD45.1/2^+^ Ly-6C^hi^ classical and Ly-6C^lo^ patrolling monocytes into naïve CD45.2^+^ recipient hosts and analyzed the percentages of donor cells in Ly-6C^lo^ patrolling monocytes, CD64^+^CD16.2^+^ monocytes and CD206^−^ IM 7 days later (Fig. 7c). Transfer of Ly-6C^lo^ monocytes, unlike that of Ly-6C^hi^ monocytes, resulted in a significant increase in the percentage of donor Ly-6C^lo^ monocytes in the lung vasculature as compared to non-transferred mice (Fig. 7d). While donor cells were hardly detectable in CD64^+^CD16.2^+^ monocytes, there was a trend towards an increase in percentages of donor cells in CD206^−^ IM when mice were transferred with Ly-6C^lo^ monocytes, but not with Ly-6C^hi^ monocytes (Fig. 7d).

In order to boost IM expansion, we injected CpG, which resulted in a drastic increase in numbers of CD64^+^CD16.2^+^ cells (Fig. 5f, g). To our surprise, we were not able to assess the chimerism of CpG-injected WT:*Nr4a1*^*−/−*^ BM mixed chimeras since they died following CpG administration, suggesting that NR4A1-dependent BM cells were needed to counteract CpG-induced toxicity (Fig. 7e). Of note, we reported a similar death in CpG-injected *Il10*^*−/−*^ mice^18^, supporting the hypothesis that CpG-induced IL-10-producing IM counteract the toxicity of CpG, and, as a corollary, that NR4A1-dependent cells could be precursors of CpG-induced IM. We injected WT, *Nr4a1*^*−/−*^, *Ccr2*^*−/−*^, and *Il10*^*−/−*^ mice with CpG and found that, while WT and *Ccr2*^*−/−*^ mice all survived after CpG injection, survival of *Nr4a1*^*−/−*^ and *Il10*^*−/−*^ mice was significantly decreased as compared to WT mice, and all surviving mice had to be euthanized after 3–6 days due to excessive weight loss (Fig. 7e). This was associated with an altered profile of CD64^+^ IM on the Ly-6C/CD64 plot and the abnormal presence of Ly-6C^+^CD64^+^ inflammatory monocytes in *Nr4a1*^*−/−*^ and *Il10*^*−/−*^ mice 3 days after CpG (Fig. 7f).

Together, our data support the notion that, at steady-state, Ly-6C^lo^ patrolling monocytes can enter the lung tissue to differentiate into CD206^−^IM via an intermediate CD64^+^CD16.2^+^ state. After CpG, NR4A1 and IL-10 are needed to counteract CpG toxicity, probably through the expansion of IL-10-producing macrophages from NR4A1-dependent precursors.

## Discussion

In this study, we investigated the diversity of lung tissue CD64-expressing mononuclear phagocytes in mice. Our study describes the existence of two main IM subpopulations in naive adult mice, confirming recent findings^22^, and further expands our knowledge about their half-life, self-maintenance potential, and localization at steady-state, as well as their stimulus-dependent regulation in an inflammatory context. In addition, we uncover that Ly-6C^lo^ patrolling monocytes can transition into extravasating CD64^+^CD16.2^+^ monocytes to give rise to CD206^−^ IM.

We have provided RNA-seq-based evidence that the CD206^+^ IM subpopulation was similar to the published Lyve-1^hi^MHC-II^lo22^ and the IM1/IM2^21^ subpopulations, whereas CD206^−^ IM profiles were similar to those of Lyve-1^lo^MHC-II^hi22^ and IM3^21^. Like Lyve-1^hi^MHC-II^lo^ IM^22^, CD206^+^ IM were larger and comprised more IL-10-expressing cells. Even though Lyve-1 was also shown to be uniquely expressed by CD206^+^ IM (Fig. 1h) and IM1/IM2^21^, the levels of Lyve-1 staining were, however, lower in CD206^+^ IM as compared to Lyve-1^hi^MHC-II^lo^ IM^22^, suggesting that CD206 may be more appropriate to discriminate IM subpopulations, at least in the lung. Conversely, CD206^−^ IM, like Lyve-1^lo^MHC-II^hi^ IM^22^, were smaller and negative for Lyve-1, and expressed higher levels of CX3CR1 and MHC-II than the other subset. Of note, two populations of tissue CD64^+^ macrophages have also been described in human lungs, one of them expressing CD206, similarly to CD206^+^ IM in mice^22^.

Regarding the maintenance of IM subsets in adults, our and others’ approaches all converged to the fact that IM subsets were slowly replaced by monocytes in adult mice^21,22^. Here, we also showed that CD206^+^ IM were able to proliferate and had an increased self-maintenance potential as compared to CD206^−^ IM^21^. Under the experimental conditions tested, we found that the half-life of CD206^−^ and CD206^+^ IM subsets were estimated at 9 and 12 months, respectively.

Quantitative data about the precise localization of lung IM (subpopulations) remained scarce and controversial^12,21,22^. In this regard, we originally observed F4/80^+^CD11c^−^ IM in the alveolar parenchyma^12^. Rodero et al. also described CX3CR1^hi^ IM in alveolar areas^37^, while Gibbings et al. proposed later that IM were uniquely located in the bronchial interstitium^21^. More recently, Chakarov et al. looked at peribronchial IM subsets and proposed that Lyve-1^lo^MHC-II^hi^ and Lyve-1^hi^MHC-II^lo^ IM were mainly associated with nerves and blood vessels, respectively^22^. To assess whether IM subsets populated distinct niches in the lung, we evaluated their preferential localization with respect to their bronchial *vs*. alveolar interstitial abundance. Our study identifies both alveolar and bronchial parenchymal IM, with CD206^+^ and CD206^−^ IM preferentially found in the bronchial and the alveolar interstitium, respectively, and bronchial CD206^+^ IM located in the vicinity of blood vessels. Additional computer-based quantitative analyses together with cutting-edge multicolor imaging technologies will likely help to unambiguously address the precise localization of lung IM subsets, at steady-state and over the course of inflammatory responses.

Importantly, IM dichotomy was also observed at the functional level. We showed that bronchial CD206^+^ IM were endowed with a superior ability to secrete immunoregulatory cytokines, including IL-10, consistent with the hypothesis that they may largely account for the reported homeostatic functions of steady-state IM^12,15^. CD206^+^ IM were also suggested to be implicated in response to wounding, which fits well with their reported role in regulating damage-induced inflammation and fibrosis^22^. Conversely, CD206^−^ IM exhibited a typical antigen-presenting cell profile and have been shown to regulate T cell-related processes^22^.

Kinetic analyses of changes in IM subsets after in vivo exposure to 3 different TLR ligands revealed a complex picture of the IM compartment during inflammation. Overall, CD64^+^CD16.2^+^ and CD206^−^ IM were more affected by the treatments than CD206^+^ IM. It is also interesting to note that LPS and CpG, which have been shown to expand IM by CCR2-dependent and independent mechanisms^18^, respectively, exhibited very distinct dynamics over time. LPS amplified the 3 IM subsets at day 3 but mostly CD206^−^ IM at day 7, while CpG robustly amplified the CD64^+^CD16.2^+^ compartment, among which most cells expressed IL-10, followed by an increase in CD206^−^ IM peaking at day 14. Moreover, there was a drop in MHC-II expression observed within CD206^−^ cells after each of the treatments, which likely reflects an influx of recruited MHC-II^lo^ monocytes into this gate, some of which giving rise later to MHC-II^hi^CD206^−^ cells (day 14). Functionally, these data suggest that CpG-elicited IM-derived protection against allergic asthma^18^ may be largely attributed to IL-10-producing CD64^+^CD16.2^+^ cells rather than CD206^−^ or CD206^+^ IM.

Besides IM subsets, we identified a discrete population of NR4A1-dependent CD64^+^CD16.2^+^ monocytes that was mainly located in the alveolar area of the lung. Such monocytes were distinct from the previously described tissue-associated Ly-6C^hi^ monocytes^18,27^ by their phenotype (CD64^+^Ly-6C^lo^CX3CR1^hi^CD16.2^+^ vs. CD64^lo^Ly-6C^hi^CX3CR1^lo^CD16.2^-^ for Ly-6C^hi^ monocytes) and their dependency on NR4A1. Using transgenic reporter mice, Rodero et al. previously detected a population of CX3CR1^hi^ monocytes located at the interface between lung capillaries and alveoli^37^. Interestingly, such monocytes were phenotypically similar to CD64^+^CD16.2^+^ monocytes and exhibited motility patterns that were distinct from those of intravascular Ly-6C^lo^ patrolling monocytes but similar to those of alveolar CX3CR1^hi^ IM^37^, consistent with our findings.

The localization, phenotype, transcriptomic profile and RNA velocity analysis of CD64^+^CD16.2^+^ monocytes, together with the mixed chimeras and adoptive transfer experiments supported the notion that CD64^+^CD16.2^+^ monocytes arose from intravascular patrolling Ly-6C^lo^ monocytes and represented an extravascular transition state towards CD206^−^ IM. First, CD64^+^CD16.2^+^ monocytes and CD206^− ^IM preferentially populated a similar micro-anatomical niche, i.e. the alveolar parenchyma^37^. Second, CD64^+^CD16.2^+^ monocytes shared similarities with patrolling Ly-6C^lo^ monocytes (i.e., surface phenotype, half-life, dependence on NR4A1). Third, despite these similarities, we observed that CD64^+^CD16.2^+^ monocytes were partly extravascular and, unlike Ly-6C^lo^ monocytes, expressed CD64 and significant levels of IL-10, a functional hallmark of lung IM^12,15,18^. Fourth, CD64^+^CD16.2^+^ monocytes upregulated many transcripts that were also highly expressed by CD206^−^ IM, suggestive of a tissue-specific imprinting. Fifth, RNA velocity analysis revealed a highly dynamic transition state of these cells, characterized by a high probability to rapidly differentiate into CD206^−^ IM on a timescale of hours^34^. Sixth, reconstitution of CD64^+^CD16.2^+^ monocytes after lethal irradiation was NR4A1-dependent, at least partially, further supporting that they derive from Ly-6C^lo^ patrolling monocytes. Seventh, some Ly-6C^lo^ patrolling monocytes that were transferred i.v. were found within the pool of CD206^−^ IM 7 days later. Finally, IM expansion induced by CpG was impaired in patrolling monocyte-deficient *Nr4a1*^*−/−*^ mice, and CpG instillation, which was well tolerated in WT or *Ccr2*^*−/−*^ mice, induced death in *Nr4a1*^*−/−*^, a phenotype also observed in *Il10*^*−/−*^ mice in which IM are not able to produce IL-10, a cytokine needed to counteract CpG toxicity.

While we have identified CD64^+^CD16.2^+^ monocytes as readily mobilizable precursors for CD206^−^ IM, our data do not rule out the possibility that they may also give rise to CD206^+^ IM, and that classical Ly-6C^hi^ monocytes could also differentiate into CD206^−^ or CD206^+^ IM, consistent with the idea that different precursors could compete for the same niche^5–8^. Of note, numbers of IM subsets are not substantially affected in *Ccr2*^*−/−*^ or *Nr4a1*^*−/−*^ mice, whose numbers of blood classical Ly-6C^hi^ and patrolling Ly-6C^lo^ monocytes are impaired, respectively^30,31^. In light of our data, it is tempting to speculate that both Ly-6C^hi^ and Ly-6C^lo^ monocytes represent potentially redundant sources for lung IM. As a corollary, Ly-6C^lo^ and Ly-6C^hi^ monocytes would represent the main source of IM subsets in *Ccr2*^*−/−*^ and *Nr4a1*^*−/−*^ mice where the respective competitors are impaired.

This is an exciting time in macrophage research. With regards to lung tissue macrophages, future efforts should be made to investigate the transcriptional programs and tissue-instructive signals tailoring the identity of IM subsets, as well as the biological functions of IM subsets in health and diseases. Novel transgenic tools allowing the selective tracking, modulation, and depletion of such cells will be instrumental in addressing these questions, and will likely have important consequences for the elaboration of therapeutic approaches for lung chronic inflammatory diseases which would target one specific IM subset while sparing the other.

## Methods

### Mice

C57BL/6J WT mice were purchased from Janvier Laboratories. *Ccr2*^*−/−*^, *Nr4a1*^*−/−*^, *Il10*^*−/−*^, WT CD45.1 and *Cx3cr1*^GFP/GFP^ (B6.129P-*Cx3cr1*^*tm1Litt*^/J) mice under the C57BL/6J background were purchased from the Jackson Laboratory (Cat. # 004999, 006187, 002251, 002014 and 005582, respectively). CD45.1/2^+^ were obtained by crossing CD45.1^+^ with CD45.2^+^ mice. IL-10-β-lactamase reporter (ITIB) C57BL/6 mice were described elsewhere^33^. *Cx3cr1*^CreERT2^.*Rosa26-LSL-YFP* C57BL/6 mice were kindly provided by Pr. G. Boeckxstaens (KU Leuven, Belgium). *Cx3cr1*^CreERT2^ mice were originally obtained from Steffen Jung, Weizmann Institute of Science^29^, and *Rosa26-LSL-YFP* mice were originally obtained from Daniel Richardson, University College London^38^. All mice were housed and bred in institutional SPF facilities at the GIGA Institute (Liège University, Belgium) and were used at 7–11 weeks of age, unless otherwise indicated. *Cx3cr1*^CreERT2^.*Rosa26-LSL-YFP* mice were bred and maintained at KU Leuven (Belgium). All animals and experimental procedures, except experiments involving *Cx3cr1*^CreERT2^.*Rosa26-LSL-YFP* mice were reviewed and approved by the Institutional Animal Care and Use Committee of the University of Liège (Belgium). Fate-mapping experiments were approved by the Animal Care and Animal Experiments Committee of the Medical Faculty of the KU Leuven (Belgium). The Guide for the Care and Use of Laboratory Animals, prepared by the Institute of Laboratory Animal Resources, National Research Council, and published by the National Academy Press, as well as European and local legislations, were followed carefully.

### Reagents and antibodies

A complete list of the reagents and antibodies used in this study can be found in Supplementary Table 3.

### Lung single cell isolation, stainings, and flow cytometry

To obtain single-lung-cell suspensions, lungs were extensively perfused with 3 ml of HBSS (Lonza) through the right ventricle, cut into small pieces with razor blades, and digested for 1 h at 37 °C in HBSS containing 5% v/v of FBS (Gibco), 1 mg.ml^−1^ collagenase A (Roche) and 0.05 mg.ml^−1^ DNase I (Roche). After 45 min of digestion, the suspension was flushed using a 18 G needle to dissociate aggregates. PBS (Gibco) containing 10 mM of EDTA (Merck Millipore) was added to stop the digestion process. The suspension was then filtered and enriched in mononuclear cells by using a density gradient (Percoll from GE Healthcare) and harvesting cells from the 1.080:1.038 g ml^−1^ interface.

Staining reactions were performed at 4 °C in FACS buffer (PBS containing 50% v/v of Brilliant Stain Buffer [BD Pharmingen], 2.5 mg ml^−1^ of BSA [Sigma], 0.5 mg ml^−1^ of sodium azide [Acros Organics]) with 2% v/v of Fc block (BD Pharmingen) to reduce non-specific binding. Cell phenotyping was performed on a FACSLSRFortessa (BD Biosciences). Cell sorting was performed on a FACSAriaIII (BD Biosciences) using the nozzle 85 at a rate allowing minimum 85% of efficiency. The purity of sorted cells was consistently above 95% for every sample. Results were analyzed using FlowJo V10 (Tree Star Inc.). Normalized MFI represents MFI for each sample with the mean of control cells MFI subtracted.

Anti-mouse CD68, Lamp-1 and Ki-67 intracellular stainings were performed by resuspending extracellular-stained cells in 500 µl of Fixation Buffer (Biolegend) for 40 min at room temperature (RT). Cells were then washed twice with 1 ml of permeabilization buffer (Thermo Fisher Scientific). Cells were stained for intracellular protein in 100 µl of fixation/permeabilization buffer (Thermo Fisher Scientific) with 2% v/v of Fc block (BD Pharmingen) for 30 min at RT.

For experiments involving ex vivo cultures and morphology assessment, cell suspensions were enriched by a magnetic-activated cell sorting (MACS) using anti-mouse CD11b microbeads (Miltenyi Biotec) according to manufacturer’s protocol, instead of the density gradient method. The negative fraction was also collected for the staining of AM.

Lung cell numbers were counted after whole lung digestion and mononuclear cell enrichment. The numbers of cells within each population were determined according to the gating strategy shown in Fig. 1a.

### Lung single cell preparation for scRNA-seq

Lung tissue CD64-expressing cells were FACS-sorted as singlet mononuclear cell-enriched CD45^+^ non-autofluorescent SSC^lo^F4/80^+^CD11c^−^Ly-6C^lo^CD64^+^ cells as shown in Fig. 1a. In the first replicate experiment, IM were isolated from lung single-cell suspensions pooled from three 10-week-old C57BL/6 female WT mice. In parallel, AM were FACS-sorted as singlet mononuclear cell-renriched CD45^+^CD11c^+^ autoflurorescent cells and were spiked in as controls at a ratio of 1:10. Such experiment was performed at KU Leuven (Belgium), while the second replicate was performed by independent experimenters through an independent pipeline using a pool of 6-month-old C57BL/6 female WT mice that were maintained in a different animal facility at the GIGA Institute (Liège University, Belgium). The 10× Genomics platform (Single Cell 3’ Solution) was used for scRNA-seq, and sequencing was performed at the Genomics Platform of the GIGA Institute (Liège University, Belgium) for both experiments. For scRNA-seq analysis of lung Ly-6C^lo^ and Ly-6C^hi^ monocytes, lung CD45^+^F4/80^+^CD11c^−^Ly-6C^lo^CD64^−^ cells and CD45^+^F4/80^+^CD11c^−^Ly-6C^hi^CD64^−^ cells were FACS-sorted, respectively. For each sample, an aliquot of Trypan blue-treated cells was examined under the microscope for counting, viability and aggregate assessment following FACS sorting. Viability was above 80% for all samples and no aggregate were observed. Cell preparations were centrifuged at 1503 RCF for 4 min and pellets were resuspended in calcium- and magnesium-free PBS containing 0.4 mg ml^−1^ of UltraPure BSA (Thermo Fisher Scientific).

Sequencing libraries were generated by using the Chromium™ Single Cell 3’ Reagent Kits v2 (10× Genomics) following the manufacturer’s instructions. GEM-RT was performed in a Veriti^©^ 96-Well Thermal Cycler (Thermo Fisher Scientific). After RT, GEMs were broken and the cDNAs were cleaned up with DynaBeads MyOne Silane beads (Thermo Fisher Scientific). cDNAs were then amplified in a Veriti^©^ 96-Well Thermal Cycler. According to the expected cell recovery (based on a 60% recovery of total loaded cells), number of amplification cycles was set to 12. Amplified cDNA products were cleaned up using SPRIselect Reagent kit (Beckman Coulter), after what purified cDNAs were quality controlled and quantified using an Agilent High Sensitivity DNA Kit (Agilent) on a 2100 Bioanalyser (Agilent). Illumina’s P5, P7 and Read2 primers, as well as Sample Index were then added to generate sequencing libraries following Chromium™ Single Cell 3’ Reagent Kits v2 protocol. Steps were as follows: (1) enzymatic fragmentation, end repair and A-tailing, (2) Double Sided Size Selection using SPRIselect reagent, (3) adaptor ligation, (4) post ligation cleanup with SPRIselect reagent, (5) Sample index PCR (number of cycles set to 14) and (6) Double Sided Size Selection using SPRIselect reagent. The barcoded sequencing libraries were quality controlled using an Agilent High Sensitivity DNA kit on a 2100 Bioanalyser and quantified by quantitative PCR (KAPA Biosystems Library Quantification Kit for Illumina platforms).

Sequencing libraries were loaded at 1.4 pM on an Illumina NextSeq500 with NextSeq 500/550 Mid Output v2 kit (150 cycles) (Illumina) using the following read lengths: 26 bp for Read1 (16 bp Barcode+10 bp Randomer), 8 bp for Sample Index and 58 bp for Read2. Cell Ranger software (v1.2.0) (10x Genomics) was used to demultiplex Illumina BCL files to FASTQ files (cellranger mkfastq), to perform alignment (to mouse GRCm38/mm10 genome), filtering, UMI counting and to produce gene—barcode matrices (cellranger count).

### Analysis of scRNA-seq samples

Analyses used R bioconductor^39^ (version 3.4.2.), and the R package Seurat^40^ (version 2.3.4).

Briefly, for analysis of lung CD64-expressing mononuclear phagocytes, we first performed a quality control analysis and selected cells for further analysis in each replicate. Cells with a minimum of 200 and a maximum of 2500 detected genes were included, and cells with more than 5% of mitochondrial genes were excluded (Supplementary Fig. 2). In addition, only genes detected in at least 3 cells were included. Gene counts were normalized by using a global-scaling method that normalizes the gene expression measurements for each cell by the total expression, multiplies it by 10,000 and log-transforms the result. The FindVariableGenes function was used to calculate highly variable genes with the x.low.cutoff, x.high.cutoff and y.cutoff parameters set to 0.0125, 3, and 0.5, respectively. Cell–cell variation in the number of detected unique molecular identifiers (UMI) was regressed out using the ScaleData function. To analyze both replicates simultaneously, we used the MergeSeurat function, creating a new object with the resulting combined data matrices and appending a given identifier to each cell name depending on which original object the cell comes from. Linear dimensional reduction was performed on the scaled data using the RunPCA function. To identify the number of statistically significant principal components (PC) to include for subsequent analyses, we used the JackStraw function, which implements a resampling test inspired by the JackStraw procedure^2^. Alternatively, we used the PCElbowPlot function, looked at a plot of the standard deviations of the PC and determined our cutoff where there is an elbow on the graph, located at ∼PC8. PC 1:8 were thus used in the subsequent analyses. We also performed analyses including lower and higher numbers of PC (1:6 to 1:12) and did not find any substantial differences in the results obtained.

The cells were clustered via the FindClusters function. Several cluster resolutions were tested, and the resolution of 0.2 was chosen, since higher resolutions created additional subdivisions or clusters containing singlets, which were considered not biologically relevant. To visualize the data, non-linear dimensional reduction was used, and *t-*SNE plots were created by using the RunTSNE function, with the number of dimensions to use set to 8 (PC 1:8). To eliminate a potential contamination with cells outside the MPS, data were subset using the SubsetData function in order to only keep cell clusters expressing detectable levels of the *Csf1r* gene.

Differential expression (DE) analysis between clusters was performed using the FindMarkers function, which uses a likelihood ratio test based on zero-inflated data to identify positive and negative markers of a single cluster compared to some or all other clusters. Only DE genes with an adjusted *P-*value < 10^−2^ were retained. To compare Cluster 3 (i.e., AM) with Clusters 1, 2, and 4 (i.e., tissue CD64-expressing cells), lists of the significantly DE genes between Cluster 3 and Clusters 1, 2, and 4 were generated. To compare the clusters of lung tissue CD64-expressing cells (i.e., Clusters 1, 2 and 4) with each other, lists of the significantly DE genes between Cluster 1 and Clusters 2 and 4, Cluster 2 and Clusters 1 and 4, or Cluster 4 and Clusters 1 and 2 were generated. Based on these lists of DE genes, Gene ontology analyses were performed using the Gene Ontology Consortium website (http://geneontology.org/) referring to the GO Ontology database released on 2018/12/01.

To visualize specific marker expression, the DotPlot function was used to show average expression of the genes and percentage of cells expressing the indicated genes within each cluster. Alternatively, the FeaturePlot or DoHeatmap functions were used to show specific gene expression across single cells.

For subsequent analyses, a similar procedure as described above was used with minor adaptations. For analysis of lung Ly-6C^lo^ and Ly-6C^hi^ monocytes, cells with a maximum of 3,000 detected genes were included, PC 1:8 were used and a resolution of 0.1 is shown for the cell clustering depicted in Supplementary Figs. 9d and 12. To compare Ly-6C^lo^ patrolling and lung CD64^+^CD16.2^+^ monocytes, data of original Cluster 4 (i.e., CD64^+^CD16.2^+^ monocytes) were subset from the dataset of lung CD64-expressing mononuclear phagocytes and merged with the data of Ly-6C^lo^ monocytes. PC 1:8 and a resolution of 0.3 were used for the cell clustering and subsequent analyses depicted in Fig. 6, Supplementary Figs. 13 and 14a, b.

For the graphical representation of RNA velocities, a Seurat object encompassing lung CD64-expressing mononuclear phagocytes, Ly-6C^lo^ patrolling monocytes and Ly-6C^hi^ classical monocytes was created and the corresponding *t-*SNE plot (PC 1:8) was used as shown in Fig. 6, Supplementary Figs. 13 and 14a, b.

### Transcriptomic comparison of lung IM subsets

To compare CD206^−^ and CD206^+^ IM subsets with Lyve1^hi^MHCII^lo^ and Lyve1^lo^MHCII^hi^ IM subsets reported in Chakarov et al.^22^, we generated CD206^−^ and CD206^+^ IM signatures (i.e., upregulated genes in CD206^−/+^ vs. CD206^+/−^ IM, respectively) based on our scRNA-seq data. Briefly, in order to be comparable between two analyses, numbers of genes within the CD206^−^ and CD206^+^ IM signatures should be similar to those of Lyve1^hi^MHCII^lo^ and Lyve1^lo^MHCII^hi^ IM signatures. Using the FindMarkers function (Seurat package^41^), we selected genes with a log fold change threshold of 0.1, and that are expressed in more than 10% of cells. The Venn diagram was generated with a publicly available online tool (http://bioinformatics.psb.ugent.be/webtools/Venn/). Gene Set Enrichment Analysis (GSEA) was performed to analyze enrichment of published Lyve1^hi^MHCII^lo^ and Lyve1^lo^MHCII^hi^ IM signatures^22^ in CD206^−^ and CD206^+^ IM (see below).

To compare CD206^−^ and CD206^+^ IM subsets with the IM1, IM2, and IM3 populations reported in Gibbings et al.^21^, data of expression counts were downloaded from Gene Expression Omnibus database (accession GSE94135) and analyzed with DESeq2 package^42^ for differential expression analysis, hierarchical clustering, and PCA analysis. As a high similarity was found between IM1 and IM2 signatures, we generated an IM1&IM2 shared signature (634 genes) and an IM3 signature (97 genes), which were used as gene sets in the GSEA analysis described below.

For GSEA, in order to analyze enrichment of published signatures in our scRNA-seq data, the normalized counts were used as expression datasets in GSEA. Briefly, genes were ranked based on their expression in CD206^+^ and CD206^−^ macrophages by Signal2Noise method. Normalized Enrichment Score (NES), FDR and nominal *p*-value were calculated with 100 permutations between samples from different phenotypes.

### Cytologic examination

Cytologic examination of FACS-sorted populations was performed on cytospin preparations stained with Hemacolor® (Merck, Cat. 111955, 111956, 111957). Sections were examined with a FSX100 microscope (Olympus) and size comparisons were performed using Image J software (NIH).

### In vivo labeling of vascular leukocytes

Lightly isoflurane-anesthetized C57BL/6J WT mice were injected i.v. with 300 µL of PBS, or with 0.5 µg of mouse APC-conjugated anti-mouse CD45.2 antibodies in 300 µl PBS. The antibodies were allowed to circulate for 3 min prior to euthanasia in order to label all leukocytes present in the vascular compartment. Lungs were harvested without perfusion and were processed for flow cytometry analysis.

### Assessment of phagocytic activity

Lightly isoflurane-anesthetized C57BL/6J WT mice were injected i.t. or i.v. with 6 × 10^8^ pHrodo™ Green *E. coli* BioParticules™ (Thermo Fisher Scientific, Cat. P35366) in 100 µl and 200 µl of PBS respectively. Lungs were harvested 3 h later for cell isolation, staining and flow cytometry analysis.

### Fate-mapping of lung CX3CR1^+^ cells

The induction of Cre recombinase to trace CX3CR1^+^ cells was performed as described elsewhere^43^. Briefly, 4 week-old *Cx3cr1*^CreERT2^.*Rosa26*-*LSL-YFP* mice were treated three times subcutaneously with 4 mg TAM (Sigma, Cat. T5648) per 30 g body weight dissolved in corn oil (Sigma, Cat. C8267), 48 h apart. The mice were sacrificed after 2, 9, 28, and 52 weeks, and lungs were processed for flow cytometry analysis. Lungs from untreated *Cx3cr1*^CreERT2^.*Rosa26-LSL-YFP* mice were used as negative control.

### Immunostainings and confocal microscopy analyses

Freshly collected lungs from C57BL/6J WT and *Cx3cr1*^GFP/GFP^ mice were embedded and frozen in OCT compound (Q Path Freeze gel, VWR), and then cut in 10 µm cryosections. Tissue sections were then fixed in 4% paraformaldehyde for 10 min at RT, permeabilized in 0.5% v/v of Triton-X100 (Sigma) for 2 min at RT and blocked in PBS containing 2% v/v of BSA and 2% of goat serum (Sigma) for 1 h at RT. Sections were first stained with a rat anti-mouse CD68 (dilution 1:100) for 2 h at RT, then with an AF568-conjugated goat anti-rat antibody (dilution 1:1000) for 2 h at RT. For samples isolated from *Cx3cr1*^GFP/GFP^ mice, sections were then stained overnight at 4 °C with AF488-conjugated antibodies against GFP (dilution 1:200) and AF647-conjugated antibodies against CD16.2 (1:20), CD206 (1:100) or MHC-II (1:50). For samples isolated from WT mice, sections were then stained overnight at 4 °C with AF647-conjugated antibodies against CD31 (1:200) or Tubb3 (1:100), and AF488-conjugated antibodies against CD16.2, CD206 or MHC-II. For assessment of MHC-II and CD206 co-expression, WT sections were simultaneously stained with antibodies against MHC-II and CD206. Cell nuclei were counterstained with 4,6-diamidino-2-phenylindole (DAPI, Biolegend) for 5 min at RT. Sections were mounted with Prolong Diamond Antifade Mountant (Thermo Fisher) and stored for at least 5 h at RT. Samples were rinsed 3 times in PBS between each of the above-mentioned steps. Images were acquired on a Zeiss LSM 880 confocal microscope (Zeiss) and analyzed using the ZEN 2.3 software (Zeiss).

Spatial distribution was quantified by analyzing, for each sample, 5 or 10 fields (magnification ×20) containing at least one CX3CR1^+^CD68^+^ cell in the peribronchial/perivascular area or the alveolar parenchyma, respectively. For each sample, the mean of the numbers of CX3CR1^+^CD68^+^CD206^+^, CX3CR1^+^CD68^+^MHC-II^+^ and CX3CR1^+^CD68^+^CD16.2^+^ cells per field was used to calculate the spatial distribution of these cells. Double positivity for CD206 and MHC-II was quantified by analyzing, for each sample, 5 fields (magnification ×20) in peribronchial/perivascular and alveolar parenchymal areas containing at least one CD68^+^CD206^+^ or CD68^+^MHC-II^+^ cell. For each sample, the numbers of CD68^+^CD206^+^, CD68^+^MHC-II^+^ cells and CD68^+^CD206^+^MHC-II^+^ cells were used to calculate the percentage of CD206/MHC-II double positive cells.

### Proteome profiler assay

CD206^−^ and CD206^+^ IM subpopulations were FACS-sorted from naive C57BL/6J WT mice and 5 × 10^4^ cells were cultured in an F-bottom 96-well plate during 16 h in 100 µl of RPMI with L-glutamine (Lonza) supplemented with 10% v/v FBS, 1% v/v MEM non-essential amino acids (Gibco), 1 mM sodium pyruvate (GE Healtcare), 50 U ml^−1^ penicillin/streptomycin (Gibco), 0.05 mM 2-mercaptoethanol (Gibco), with or without 10 ng ml^−1^ of LPS (Sigma). For each experimental condition, supernatants were tested for the presence of cytokines and chemokines using a proteome profiler mouse XL cytokine array (R&D Systems), according to manufacturer instructions. Results were visualized using an ImageQuant LAS 4000 (GE Healthcare) and analyzed using ImageJ software. Results are expressed as Z-scores for each analyte ([individual value – analyte mean] per analyte standard deviation).

### Assessment of IL-10 expression in ITIB mice

To assess IL-10 expression using IL-10-β-lactamase reporter ITIB mice^33^, lung cells from IL-10-β-lactamase reporter ITIB and control WT mice were resuspended in a CCF4-AM (Thermo Fisher Scientific, Cat. K1028)-containing solution supplemented with probenecid (Thermo Fisher Scientific, Cat. P36400) prepared according to manufacturer’s instructions, and incubated 90 min at 29 °C. CCF4-loaded cells were then classically stained and analyzed by flow cytometry. CCF4-loaded cells from C57BL/6J WT mice were used as negative controls.

### Estimations of RNA velocities in single cells

RNA velocities in single cells were estimated with RNA velocity (http://velocyto.org)^34^. Briefly, spliced and non-spliced transcripts counts were calculated from CellRanger output using Python command line tool velocyto with default run10x subcommand. Genes with minimum average expression of 0.5 (for spliced transcripts) or 0.05 (for non-spliced transcripts) within at least one cell cluster were filtered before velocity estimation. RNA velocities were estimated using 20 k-nearest neighbors (NN) in slope calculating smoothing, and fit quantile of 0.02. RNA velocities were then visualized using correlation-based transition probability matrix within the kNN graph, with same cluster labels and embedding as in Fig. 6a.

To compare RNA velocity across subsets of Ly-6C^lo^ patrolling monocytes, CD64^+^CD16.2^+^ monocytes and CD206^−^ IM, the relative distance from the cell position in the 2D-tSNE plot to the projected position (i.e., effective length of arrows displayed in Supplementary Fig. 13b) was used to illustrate the RNA velocity of each cell.

### Cell fate decision estimation

Cell fate decision was represented by RNA-velocity-based single-step transition probabilities from starting cells to neighboring cells. To illustrate cell fate decision probabilities of all cells in a given subset (i.e., the starting subset), the total transition probability (TP*n*) to neighboring cells *n* was calculated as the sum of all transition probabilities (TP*in*) of single cells in the given subset (containing *j* cells), and corrected by the number of cells:

$${\mathrm{TP}}n = \frac{{\mathop {\sum }\nolimits_{i = 1}^j {\mathrm{TP}}in}}{j}$$

For illustration, transition possibility of each cell position was indicated by a color gradient while an ellipse marked the starting subset of interest with 95% confidence on the cell positions in the subset.

### Trajectory analyses

For trajectory analyses, the previously published package Slingshot^35^ was used. Briefly, Slingshot uses pre-existing clustering information to calculate development trajectory and pseudo-time of each cell in the development trajectory. To analyze the differentiation trajectory from Ly-6C^lo^ patrolling monocytes to CD206^−^ IM subpopulation, we reanalyzed the data sets shown in Fig. 6a with Seurat using higher resolution (i.e., 2.5), and the 5 clusters covering Ly-6C^lo^ patrolling monocytes, CD64^+^CD16.2^+^ monocytes, and neighboring CD206^−^ IM were used for Slingshot analysis. For clarity purposes, the original *t-*SNE embedding of Fig. 6a was retained to illustrate cell positions in Fig. 6e and the ellipses with 80% confidence were drawn to illustrate their cluster belonging shown in Fig. 6a using the same colors. To find the temporally expressed genes through trajectory, we used the method suggested in the Slingshot package to regress genes on the pseudo-time variable using a general-additive model. Expression of the top 100 genes on *p*-value was showed in a heatmap across all cells in selected subsets.

### White blood cell isolation

To obtain single-blood-cell suspensions, blood was collected in a tube containing 500 µL of PBS (Gibco) containing 100 mM of EDTA (Merck Millipore) and red blood cells were lysed by adding distilled water containing 150 mM of ammonium chlorure (VWR) and 10 mM of potassium bicarbonate (Sigma). Cells were then centrifuged, resuspended in FACS buffer and stained as described for lung cells in the respective section above.

### Bone marrow mixed chimeras

Eighteen-week-old CD45.1/2+WT mice were lethally irradiated with two doses of 6 Gy 3 h apart. Two hours after the second irradiation, these mice were injected i.v. with 2 × 10^6^ BM cells consisting of a 1:1 mix of BM cells obtained from CD45.1^+^ WT and CD45.2^+^ *Nr4a1*^*−/−*^ mice. From the day before irradiation, mice were treated for 4 weeks with 0.05 mg.ml^−1^ of enrofloxacin (Baytril, Bayer). Chimerism was assessed by flow cytometry in the blood and the lung 6 and 14 weeks after irradiation, respectively. In the blood, B lymphocytes, Ly-6C^lo^ monocytes, Ly-6C^hi^ monocytes and neutrophils, were defined as CD45^+^ CD11b^−^Ly-6G^−^CD19^+^, CD45^+^CD11b^+^Ly-6G^−^CD115^+^Ly-6C^−^, CD45^+^CD11b^+^Ly-6G^−^CD115^+^Ly-6C^+^ and CD45^+^CD11b^+^Ly-6G^+^ cells, respectively.

### Monocyte adoptive transfers

Blood cells of CD45.1/2^+^ mice were collected and stained for FACS sorting of Ly-6C^hi^ and Ly-6C^lo^ monocytes, defined as CD45^+^CD11b^+^CD115^+^Ly-6C^+^ and CD45^+^CD11b^+^CD115^+^Ly-6C^+^ cells, respectively. These cells were then transferred to CD45.2^+^ WT recipient mice by injecting 10^5^ Ly-6C^hi^ or Ly-6C^lo^ monocytes i.v. into each mouse. Lungs were harvested 7 days later for cell isolation, staining and flow cytometry analysis.

### Intratracheal instillations of TLR ligands

Lightly isoflurane-anesthetized C57BL/6J WT mice were injected i.t. with Pam3CSK4, LPS or CpG (25 µg, 10 µg and 50 µg, respectively) in 50 µl of PBS. Lungs were harvested 1, 3, 5, 7, 14 or 28 days later for cell isolation, staining and flow cytometry analyses. C57BL/6 WT, *Ccr2*^*−/−*^, *Nr4a1*^*−/−*^, *Il10*^*−/−*^, and WT:*Nr4a1*^*−/−*^ 1:1 BM mixed chimeras were injected i.t. with 50 µg of CpG in 50 µl of PBS. The survival of these mice was assessed and lungs from WT, *Nr4a1*^*−/−*^ and *Il10*^*−/−*^ mice were harvested 3 days later for flow cytometry analysis. ITIB mice were injected i.t. with 50 µg of CpG in 50 µl of PBS and their lungs were harvested 7 days later for cell isolation, staining and IL-10 production analysis by flow cytometry.

### Statistical analysis

Data from independent experiments were pooled for analysis in each data panel, unless otherwise indicated. Statistical analyses were performed using Prism 7 (GraphPad Software), SAS (version 9.3) and R bioconductor^39^ (version 3.4.2.). Data were presented as mean ± s.e.m., as well as individual values, unless otherwise indicated. We considered a *P-*value lower than 0.05 as significant. *P < 0.05; **P < 10^−2^, ***P < 10^−3^; ****P < 10^−4^; ns, not significant. Details about the statistical tests used can be found in the respective Figure legends. Details about the analysis of scRNA-seq data can be found in the respective sections above.

### Reporting summary

Further information on research design is available in the Nature Research Reporting Summary linked to this article.

## Supplementary information

Supplementary Information

Reporting Summary

Source Data

## Data Availability

The scRNA-seq data provided in this manuscript have been deposited in the ArrayExpress database at EMBL-EBI under accession number E-MTAB-7678. The source data underlying Figs. 1f, g, j; 2b, c, f and h; 3c–f; 4b; 5d, f, h and i; and 7b, d, e and Supplementary Figs. 7c; 8 b, d; 9b; and 10 are provided as a Source Data file, as mentioned in the respective Figure legends.
