# Supplementary material for: Non-classical tissue monocytes and two functionally distinct populations of interstitial macrophages populate the mouse lung
Source: Nat Commun. 2019 Sep 3;10:3964. doi: 10.1038/s41467-019-11843-0 (PMC6722135; doi:10.1038/s41467-019-11843-0)
Supplement: Supplementary file 1 — Supplementary Information [file 41467_2019_11843_MOESM1_ESM.pdf]

Non-classical tissue monocytes and two functionally distinct populations of interstitial macrophages populate the mouse lung

Supplementary information

Joey Schyns[1,2,3], Qiang Bai[1,3], Cecilia Ruscitti[1,3], Coraline Radermecker[1,3], Sebastiaan De Schepper[4], Svetoslav Chakarov[5], Frédéric Farnir[3,6], Dimitri Pirottin[1,3], Florent Ginhoux[5,7], Guy Boeckxstaens[4], Fabrice Bureau[2,3,8], Thomas Marichal[1,2,3,8,*]

Laboratories of [1] Immunophysiology and [2] Cellular and Molecular Immunology and GIGA Institute, Liège University, Avenue de l'Hôpital 11, B34, 4000 Liège, Belgium.
[3] Faculty of Veterinary Medicine, Liège University, Boulevard de Colonster 20, 4000 Liège, Belgium.
[4] Translational Research Center for Gastrointestinal Disorders, Department of Chronic Diseases, Metabolism and Ageing, KU Leuven, Herestraat 49, O&N1, Box 701, 3000 Leuven, Belgium.
[5] Singapore Immunology Network (SIgN), A*STAR, Biomedical Grove 8a, 138648 Singapore.
[6] FARAH Institute, Liège University, Boulevard de Colonster 20, 4000 Liège, Belgium.
[7] Shangai Institute of Immunology, Shanghai JiaoTong University School of Medicine, South Chongqing Road 280, 200002 Shanghai, China
[8] WELBIO, Walloon Excellence in Life Sciences and Biotechnology, Wallonia, Belgium.

*** Correspondence**
Dr. Thomas Marichal, DVM, PhD
Laboratory of Immunophysiology,
GIGA Institute, Liège University,
Quartier Hôpital, B34
Avenue de l'Hôpital, 11, 4000 Liège
+32 (0) 4 / 366 95 55
t.marichal@uliege.be

Supplementary Figures

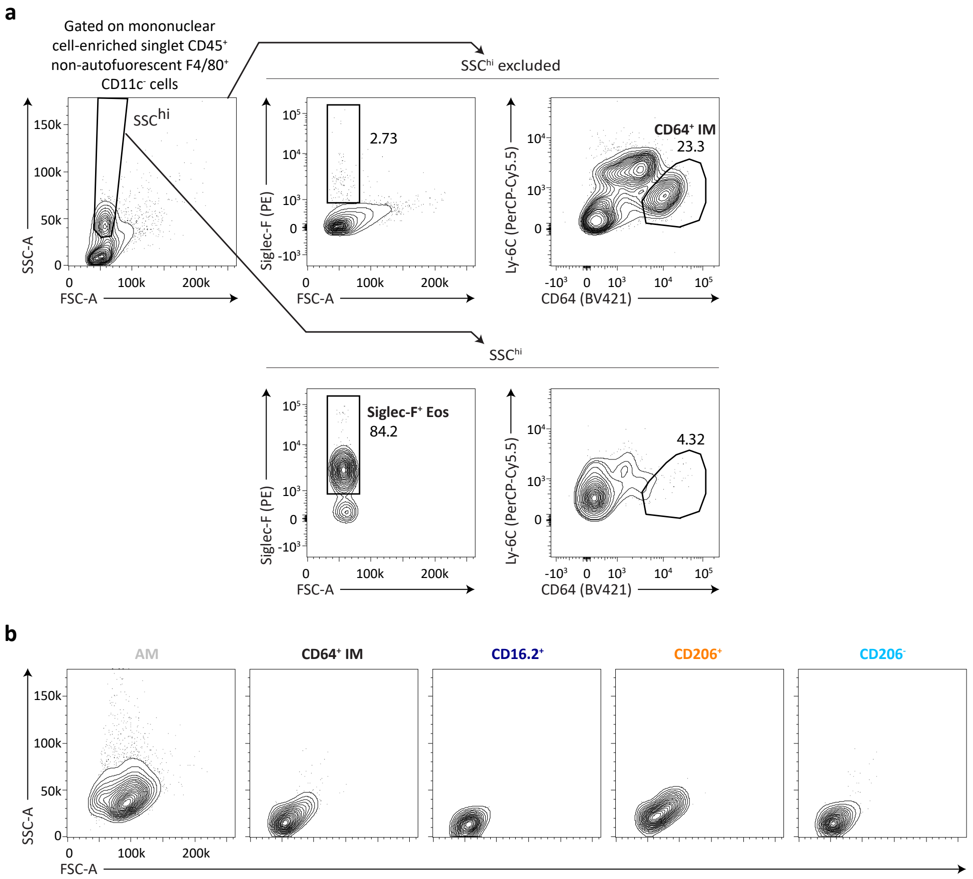

Supplementary Figure 1 Validation of the gating strategy used to delineate AM and IM subsets (see Fig. 1a). **a** Siglec-F staining was used to confirm that eosinophils were efficiently excluded without substantial loss of $CD64^+$ IM. Representative contour plots of the indicated markers are shown for (top) SSC^{hi}-excluded cells or (bottom) SSC^{hi} cells. Numbers indicate the percentage of cells within the indicated gate. **b** FSC/SSC back-gating of AM, $CD64^+$ IM (as shown in Fig. 1a) and IM subsets (as shown in Fig. 1e).

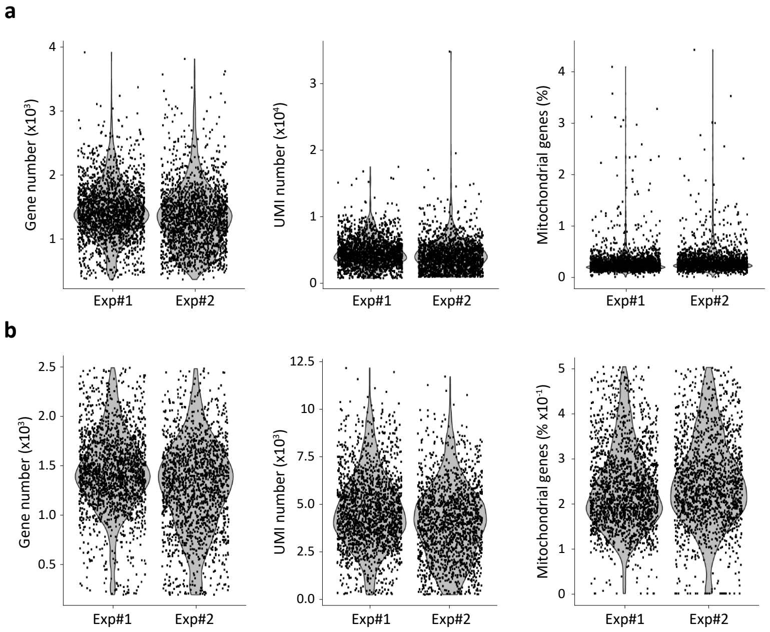

Supplementary Figure 2 Homogeneity in the numbers of detected genes and UMIs, and low percentage of mitochondrial genes within the independent replicates analyzed by scRNA-seq in Figure 1. (**a,b**) Gene numbers (left), Unique Molecular Identifiers (UMI) numbers (middle) and percentage of mitochondrial genes (right) detected in single cells from the indicated groups (**a**) before and (**b**) after selection and filtering, presented as violin plots (height: gene numbers [left], UMI numbers [middle] and % Mitochondrial genes [right]; width: abundance of cells) and individual dots representing individual cells. Exp, experiment.

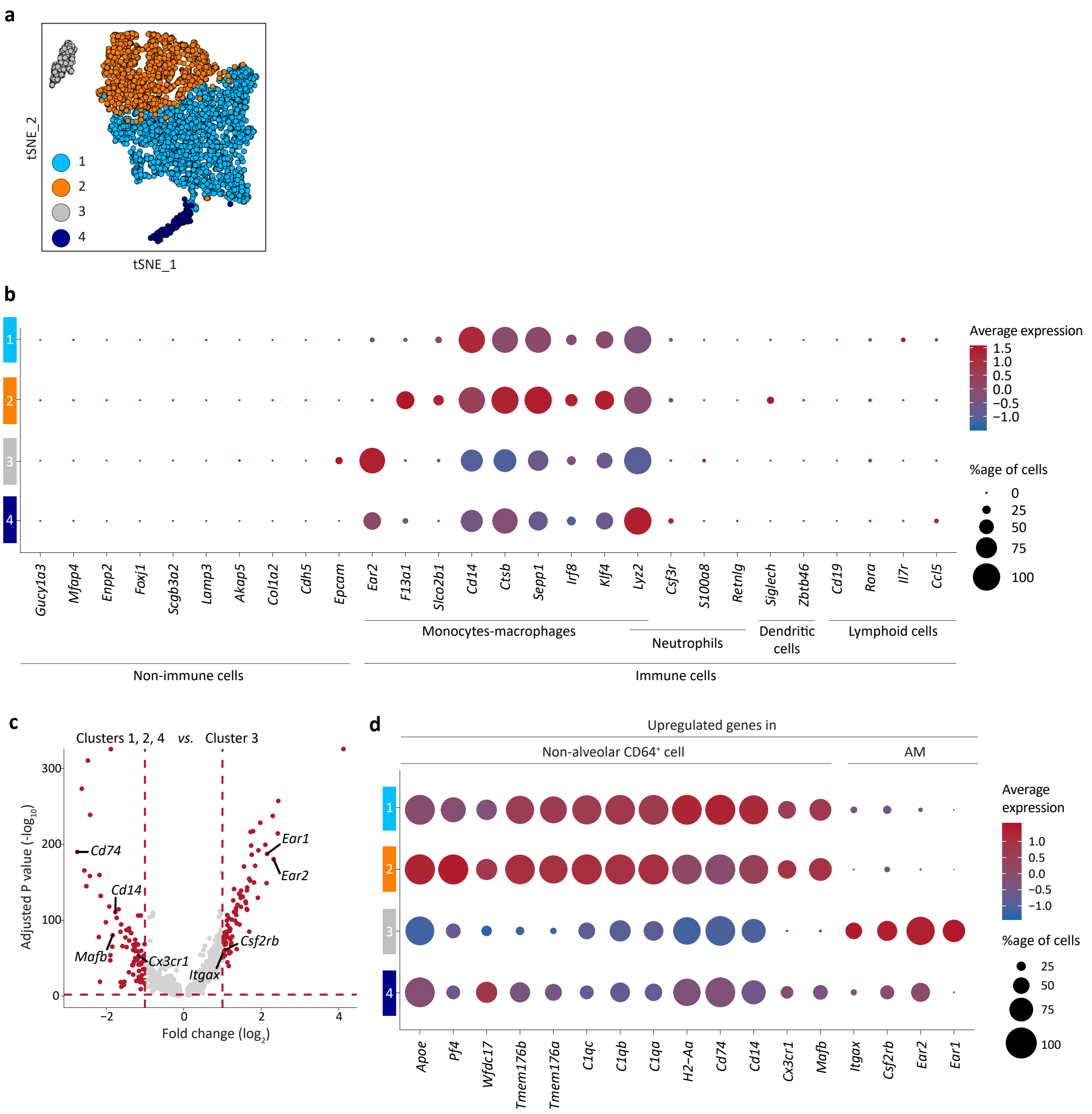

Supplementary Figure 3 The lung CD64$^+$ cells analyzed by scRNA-seq were identified as monocytes/macrophages. **a** *t*-SNE plot depicting the cell clusters analyzed by scRNA-seq in Figure 1. **b** Dot plots showing average expression of genes within each cluster. We ruled out any contamination by non-immune cells, lymphoid cells, neutrophils or dendritic cells (DC) and confirmed a high expression of prototypical markers of the MPS, such as *Cd14*, *Ctsb*, *Irf8* and *Klf4*[1,2]. **c** Volcano plot representation of the differentially expressed genes between Clusters 1, 2 and 4 (i.e., non-alveolar CD64$^+$ cells) and Cluster 3 (i.e., AM). Genes with an adjusted *P* value (P_{adj})<10^{-2} and a biological FC >2 are colored in red. A total of 1,332 genes were differentially expressed between Cluster 3 (AM) and Clusters 1, 2 and 4 (P_{adj}< 10^{-2}). **d** Dot plots showing average expression of genes within each cluster. Examples of genes differentially regulated (P_{adj}<10^{-2}) between non-alveolar CD64$^+$ cells and AM are depicted. Cluster 3 was characterized by high expression of *Itgax* (encoding CD11c), *Csf2rb, Ear1*, *Ear2,* thus representing AM[3]. AM, alveolar macrophage; FC, fold change.

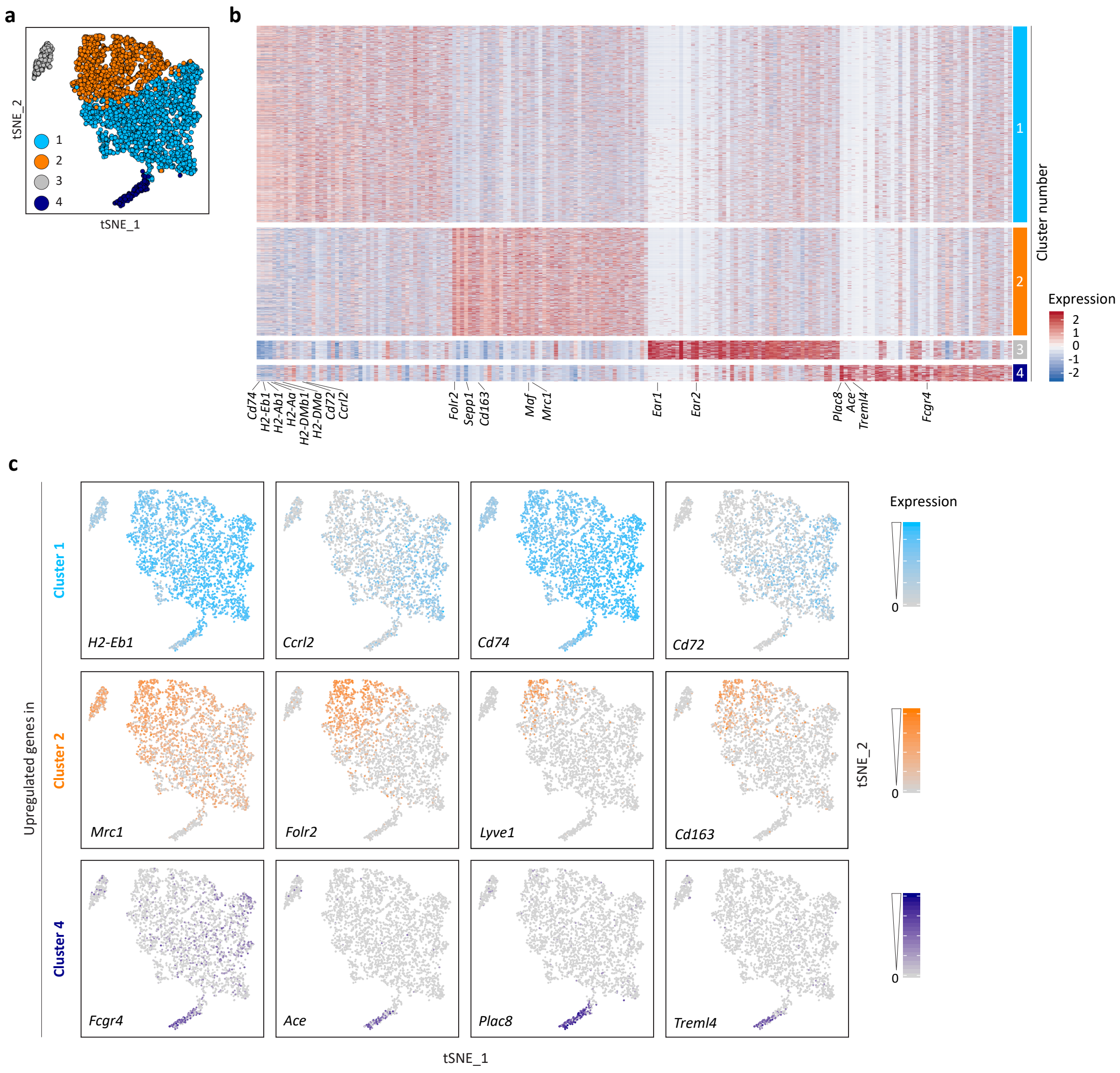

Supplementary Figure 4 Single cell expression of cluster-related genes. **a** *t*-SNE plot depicting the cell clusters analyzed by scRNA-seq in Figure 1. **b** Heatmap depicting the single-cell expression of the 50 most upregulated genes across identified clusters shown in the *t*-SNE plot in **a**. Inserts indicate the name of individual genes. **c** *t*-SNE plot showing single cell expression of the genes depicted in Fig. 1d.

Comparison of CD206- and CD206+ IM with Lyve1^{lo}MHCII^{hi} and Lyve1^{hi}MHCII^{lo} IM (Chakarov et al., *Science* 363 (6432), 2019)

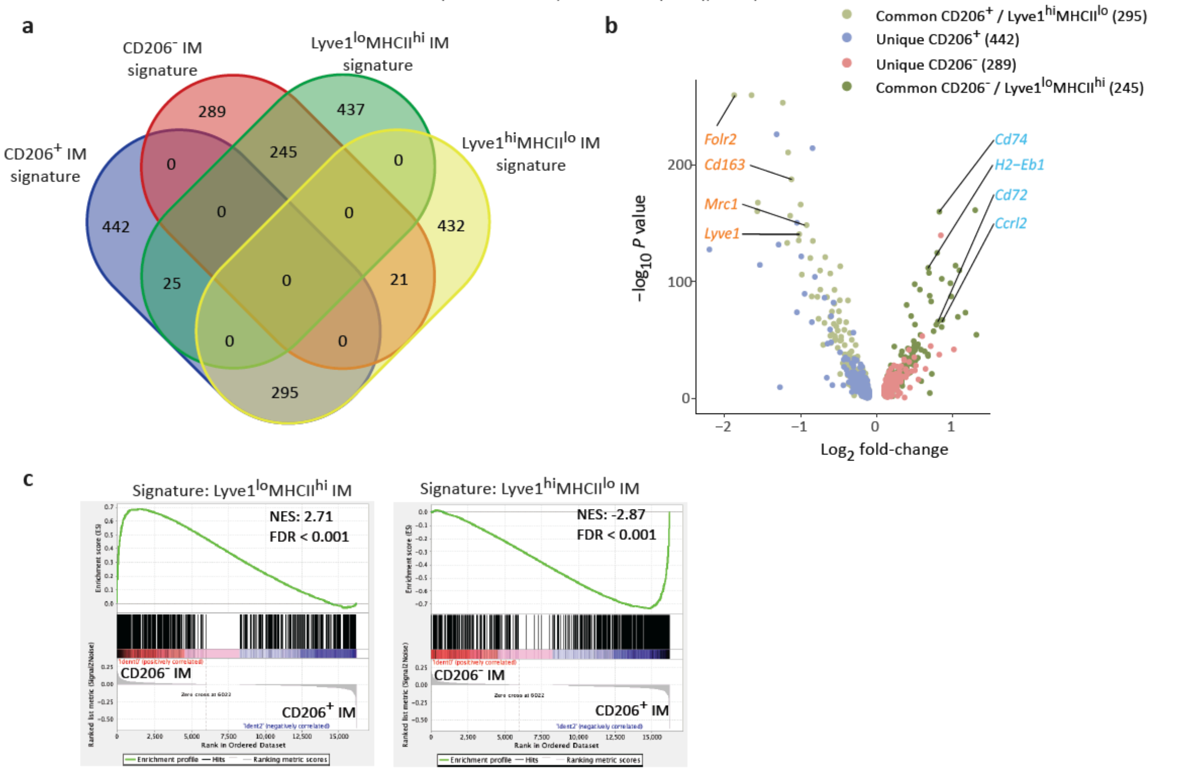

Comparison of CD206- and CD206+ IM with IM1, IM2 & IM3 (Gibbings et al., *Am J Resp Cell Mol Biol* 57, 66-76, 2019)

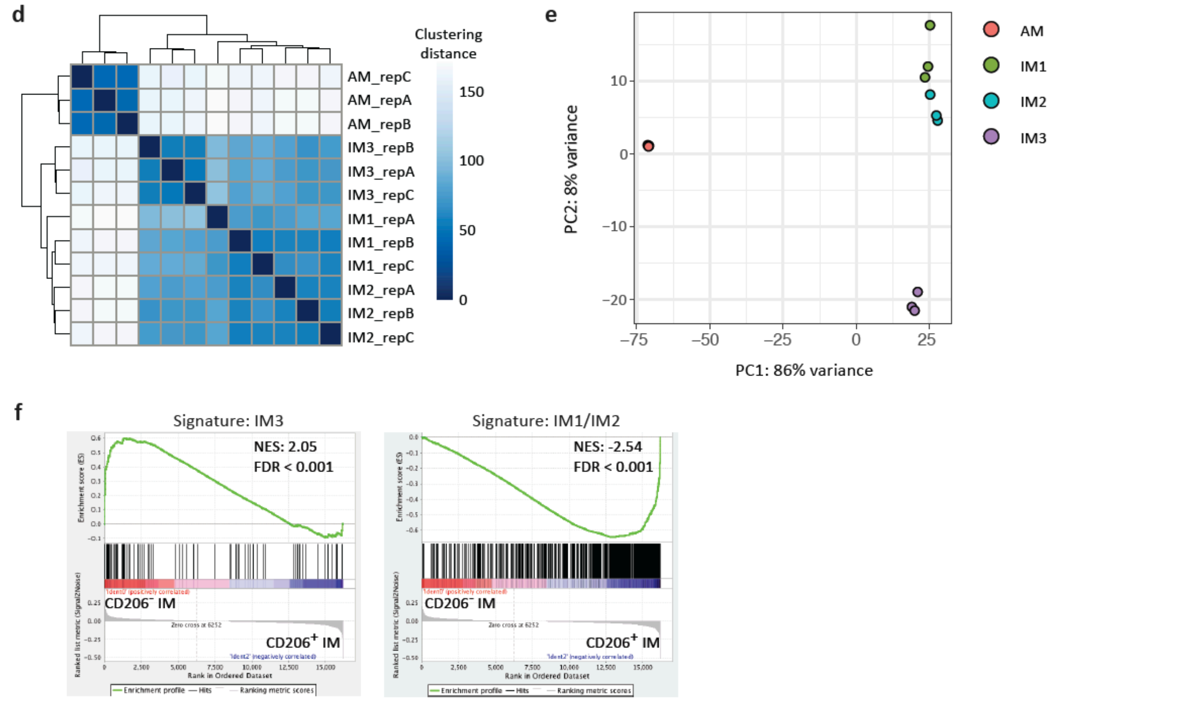

Supplementary Figure 5 Transcriptomic comparison of $CD206^+$ and $CD206^-$ IM subsets with previously described IM subsets. (**a-c**) Comparison of $CD206^+$ and $CD206^-$ IM with $Lyve1^{hi}MHCII^{lo}$ and $Lyve1^{lo}MHCII^{hi}$ IM[4] **a** Venn Diagram detailed overlap of DE genes between all 4 IM subpopulations. **b** Volcano plot representation of the genes belonging to the $CD206^+$ and $CD206^-$ IM signatures and discriminated between 'common' *vs.* 'unique' as compared with $Lyve1^{hi}MHCII^{lo}$ and $Lyve1^{lo}MHCII^{hi}$ IM signatures[4], respectively. **c** GSEA plots showing comparisons between $CD206^+$ and $CD206^-$ IM profiles and $Lyve1^{hi}MHCII^{lo}$ and $Lyve1^{lo}MHCII^{hi}$ IM signatures[4]. (**d-f**) Comparisons of $CD206^+$ and $CD206^-$ IM with IM1, 2 and 3[5]. **d** Unsupervised hierarchical clustering of biological replicates of AM, IM1, 2 and 3. **e** Two-dimensional PCA comparing AM, IM1, 2 and 3. Percentages indicate the variability explained by each component. **f** GSEA plots showing comparisons of $CD206^+$ and $CD206^-$ IM profiles with IM1/2 and IM3 signatures[4]. DE, differentially expressed; FDR, false discovery rate; GSEA, Gene Set Enrichment Analysis; NES, normalized enrichment score; PC(A), Principal Component (Analysis).

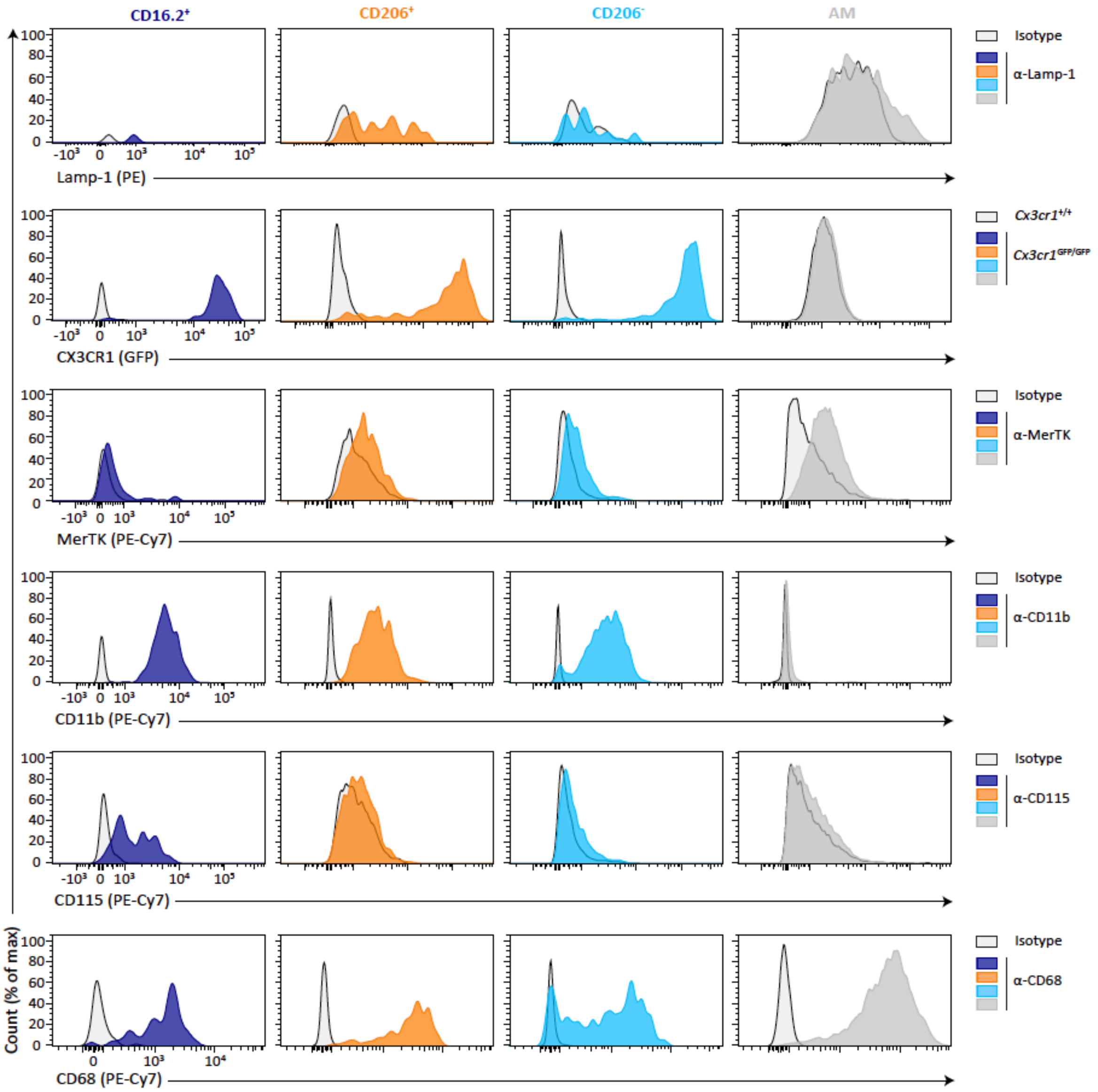

Supplementary Figure 6 Immunophenotyping of lung CD16.2$^+$ monocytes and IM subpopulations. Representative histograms of the indicated markers are shown, whose quantification is provided in Fig. 2c. *Cx3cr1*$^{+/+}$ or isotype-stained cells were used as controls.

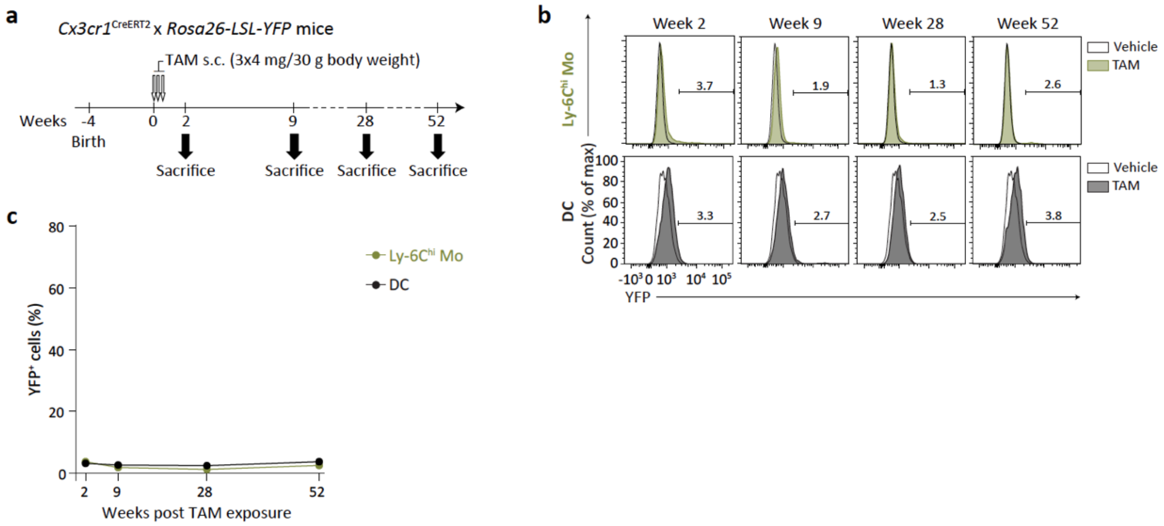

Supplementary Figure 7 Virtual absence of YFP labeling in Ly-6C^{hi} classical monocytes and lung DC 2 weeks after TAM exposure in *Cx3cr1*^{CreERT2}.*Rosa26-LSL-YFP* mice. **a** Experimental outline for panels **b, c**. Briefly, at 4 weeks of age, *Cx3cr1*^{CreERT2}.*Rosa26-LSL-YFP* mice were treated with TAM s.c. 3 times, 48h apart. Mice were analyzed for YFP expression 2, 9, 28 and 52 weeks later. **b** Representative histograms of YFP expression within the indicated populations (also see Supplementary Table 1 for gating strategies). Numbers indicate the percentage of YFP^{+} cells, as quantified in **c**. **c** Percentage of YFP^{+} cells within the indicated populations, assessed by flow cytometry. Data show mean ± s.e.m. and are pooled from 2 independent experiments (n=10). Source data are provided as a Source Data file. DC, dendritic cell; s.c., subcutaneous; TAM, tamoxifen.

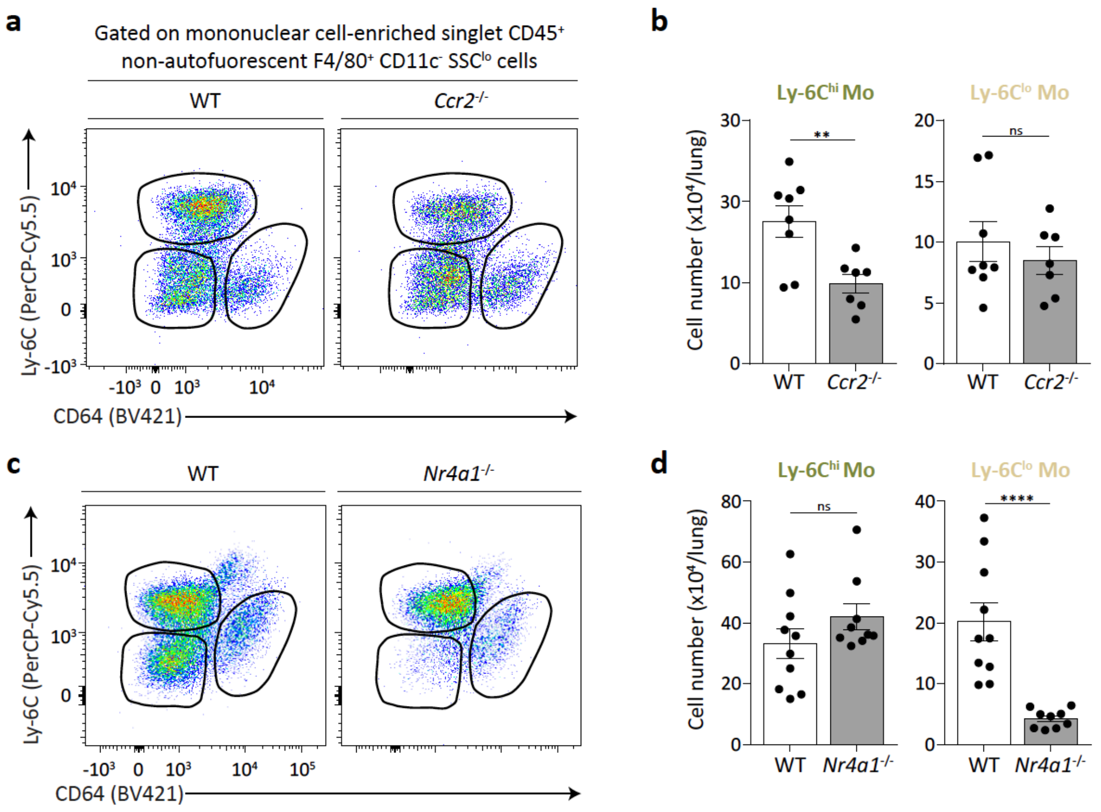

Supplementary Figure 8 Numbers of lung Ly-6C^{hi} and Ly-6C^{lo} monocytes in *Ccr2*$^{-/-}$, *Nra41*$^{-/-}$ and WT control mice. (**a, c**) Representative dot plots of Ly-6C and CD64 expression gated on mononuclear cell-enriched singlet CD45^{+} non-autofluorescent F4/80^{+}CD11c^{-}SSC^{lo} cells and discriminating lung Ly-6C^{hi} classical, Ly-6C^{lo} patrolling monocytes and bulk IM in (**a**) *Ccr2*$^{-/-}$, (**c**) *Nr4a1*$^{-/-}$ and corresponding control mice. (**b, d**) Absolute numbers of the indicated cell populations in the lungs of (**b**) *Ccr2*$^{-/-}$ or (**d**) *Nr4a1*$^{-/-}$ and control WT mice. Data show mean ± s.e.m., as well as individual mice (n=7-10/group), and are pooled from 2-3 independent experiments. *P* values were calculated using a two-tailed unpaired Student's *t* test. Source data are provided as a Source Data file. **, $P<10^{-2}$; ***, $P<10^{-3}$; ns, not significant.

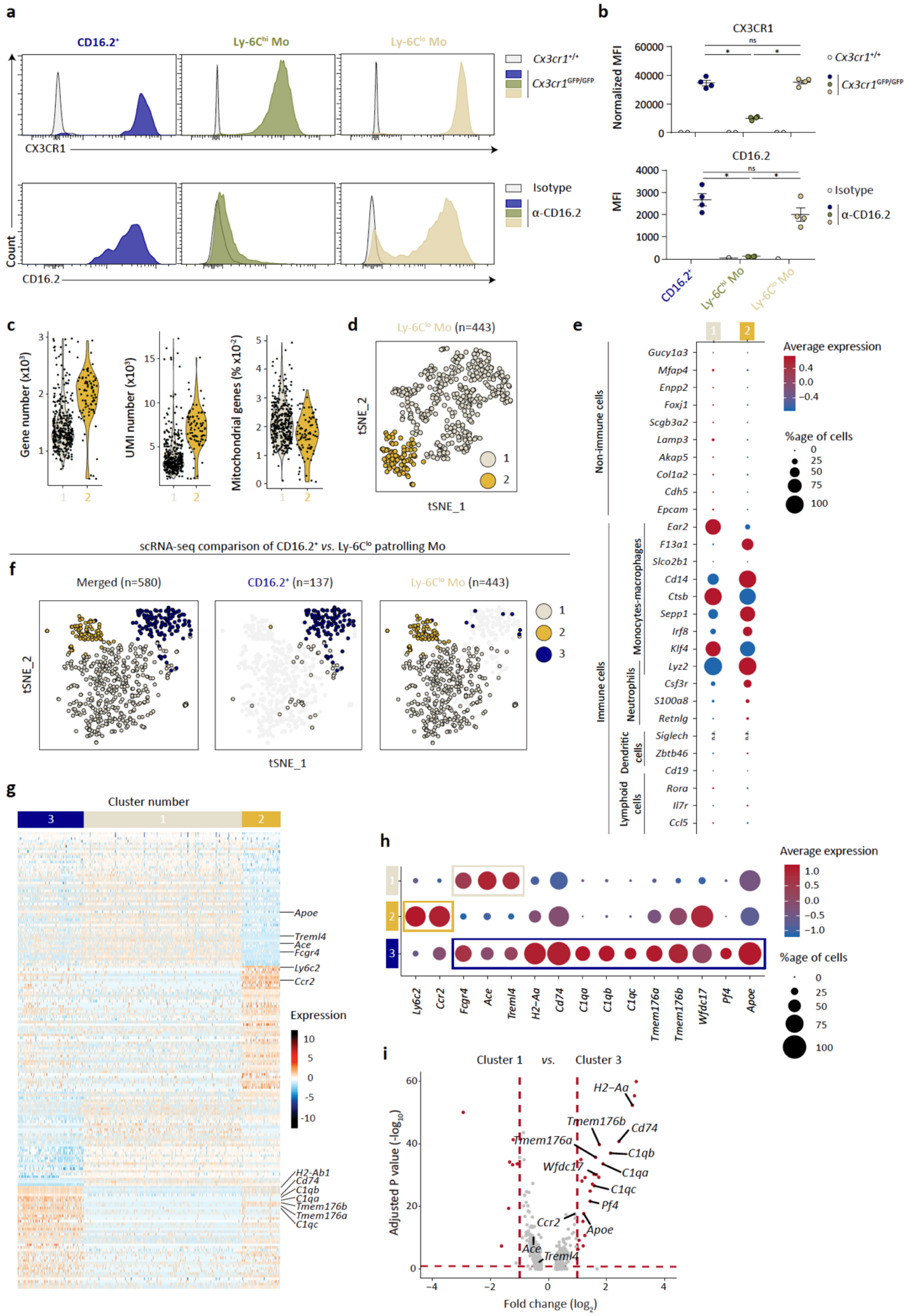
a
CD16.2+
Ly-6Chi Mo
Ly-6Clo Mo
Cx3cr1+/+
Cx3cr1GFP/GFP
CX3CR1
Isotype
α-CD16.2
Count
CD16.2
b
CX3CR1
Normalized MFI
CD16.2
MFI
ns
CD16.2+
Ly-6Chi Mo
Ly-6Clo Mo
c
Gene number (x10³)
UMI number (x10³)
Mitochondrial genes (% x10⁻²)
d
Ly-6Clo Mo (n=443)
tSNE_2
tSNE_1
e
Average expression
%age of cells
Non-immune cells
Immune cells
Monocytes-macrophages
Neutrophils
Dendritic cells
Lymphoid cells
Gucy1a3
Mfap4
Enpp2
Foxj1
Scgb3a2
Lamp3
Akap5
Col1a2
Cdh5
Epcam
Ear2
F13a1
Slco2b1
Cd14
Ctsb
Sepp1
Irf8
Klf4
Lyz2
Csf3r
S100a8
Retnlg
Siglech
Zbtb46
Cd19
Rora
Il7r
Ccl5
scRNA-seq comparison of CD16.2+ vs. Ly-6Clo patrolling Mo
f
Merged (n=580)
CD16.2+ (n=137)
Ly-6Clo Mo (n=443)
g
Cluster number
Expression
Apoe
Treml4
Ace
Fcgr4
Ly6c2
Ccr2
H2-Ab1
Cd74
C1qb
C1qa
Tmem176b
Tmem176a
C1qc
h
Average expression
%age of cells
Ly6c2
Ccr2
Fcgr4
Ace
Treml4
H2-Aa
Cd74
C1qa
C1qb
C1qc
Tmem176a
Tmem176b
Wfdc17
Pf4
Apoe
i
Cluster 1 vs. Cluster 3
Adjusted P value (-log10)
Fold change (log2)
H2-Aa
Tmem176b
Cd74
Tmem176a
C1qb
Wfdc17
C1qa
C1qc
Pf4
Ccr2
Apoe
Ace
Treml4

Supplementary Figure 9 Similarities and differences between lung CD64$^+$CD16.2$^+$ and Ly-6C^{lo} patrolling monocytes. (**a,b**) CX3CR1 and CD16.2 expression on lung CD64$^+$CD16.2$^+$ monocytes, Ly-6C^{hi} classical and Ly-6C^{lo} patrolling monocytes. **a** Representative histograms of the indicated markers. *Cx3cr1*$^{+/+}$ or isotype-stained cells were used as controls. **b** Quantification of expression of the indicated markers as compared to control cells. Data show mean ± s.e.m., as well as individual mice (n=2-4/group). *P* values were calculated using a two-way ANOVA with Tukey's post hoc test. (**c-e**) scRNA-seq analysis of lung Ly-6C^{lo} patrolling monocytes. **c** Gene numbers (left), Unique Molecular Identifiers (UMI) numbers (middle) and percentage of mitochondrial genes (right) detected in single cells from each cluster after selection and filtering, presented as violin plots (height: gene numbers [left], UMI numbers [middle] and % Mitochondrial genes [right]; width: abundance of cells) and individual dots representing individual cells. **d** *t*-SNE plot depicting the single cells analyzed by scRNA-seq. **e** Dot plots showing average expression of genes characteristic of the indicated cell types within each cluster. (**f-i**) Comparison of lung CD64$^+$CD16.2$^+$ monocytes and Ly-6C^{lo} patrolling monocytes by scRNA-seq. **f** From left to right: *t*-SNE plot from the merged conditions, from CD64$^+$CD16.2$^+$ monocytes (i.e., Cluster 4 of Fig. 1c) or from Ly-6C^{lo} patrolling monocytes are shown. *n* indicates the number of cells analyzed after quality control and filtering. **g** Heatmap depicting the single-cell expression of the most upregulated genes across identified clusters. Inserts indicate the name of individual genes. **h** Dot plots showing average expression of genes within each cluster. Cluster 2 expressed high levels of the classical monocyte markers *Ly6c2* and *Ccr2* when compared to Clusters 1 and 3, which were both characterized by a greater expression of *Fcgr4, Ace* and *Treml4,* supporting that Cluster 3 comprised Ly-6C^{lo} monocytes that recently arose from Ly-6C^{hi} classical monocytes and retained mRNA expression of *Ly6c2* and *Ccr2*. **i** Volcano plot representation of the differentially expressed genes between Cluster 1 (i.e., 'mature' Ly-6C^{lo} patrolling monocytes) and Cluster 3 (i.e., CD64$^+$CD16.2$^+$ monocytes) are shown. Genes with a $P_{adj}<10^{-2}$ and a biological FC >2 are colored in red. Inserts indicate the names of individual genes. Source data are provided as a Source Data file. *, $P<0.05$; ns, not significant. MFI, Mean Fluorescence Intensity.

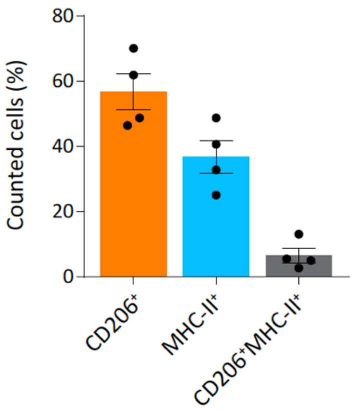

Supplementary Figure 10 Evaluation of MHC-II$^+$CD206$^+$ double-positive cells in high-resolution confocal microscopy pictures of lung sections. Lung sections of C57BL/6 WT mice were assessed for the presence of MHC-II$^+$CD206$^+$ double-positive cells. Bar graph shows the % of CD206$^+$MHC-II$^-$, CD206$^-$MHC-II$^+$ and CD206$^+$MHC-II$^+$ IM among counted cells. Data shown mean ± s.e.m., as well as individual analyzed mice (n=4).

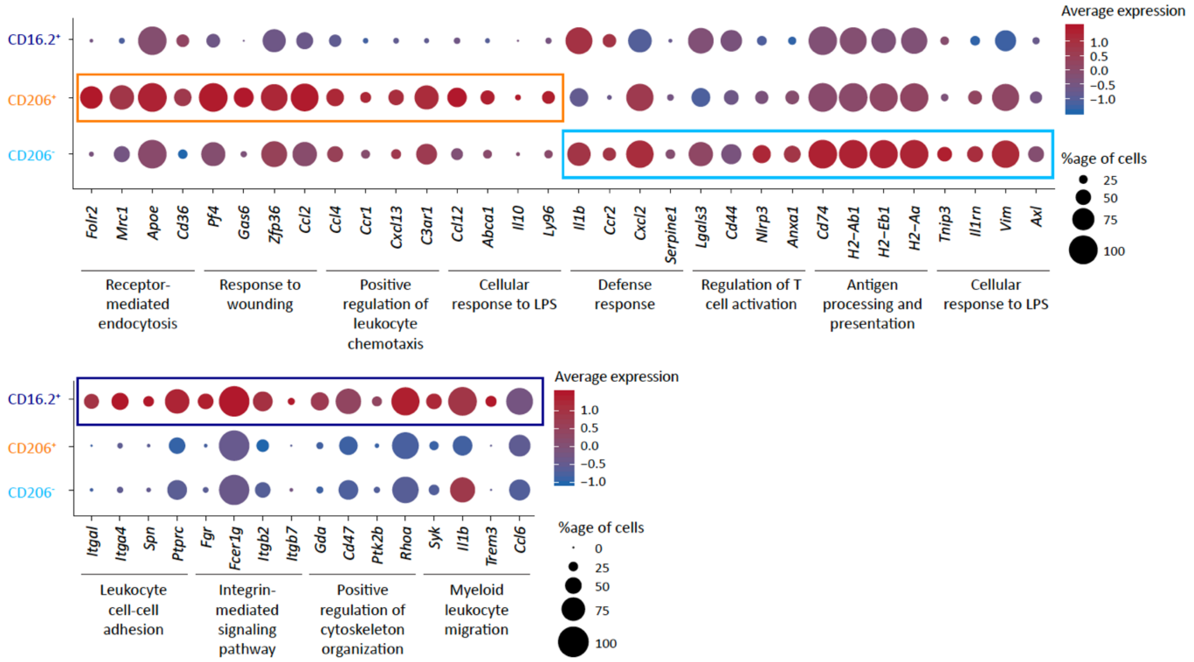

Supplementary Figure 11 Functional properties of CD206+ and CD206- IM subsets and CD64+CD16.2+ monocytes based on scRNA-seq and Gene Ontology (GO) analyses. Dot plots showing average expression of the indicated genes and percentage of cells expressing the genes within each cluster. Examples of genes involved in the biological responses shown in Table 1 are shown.

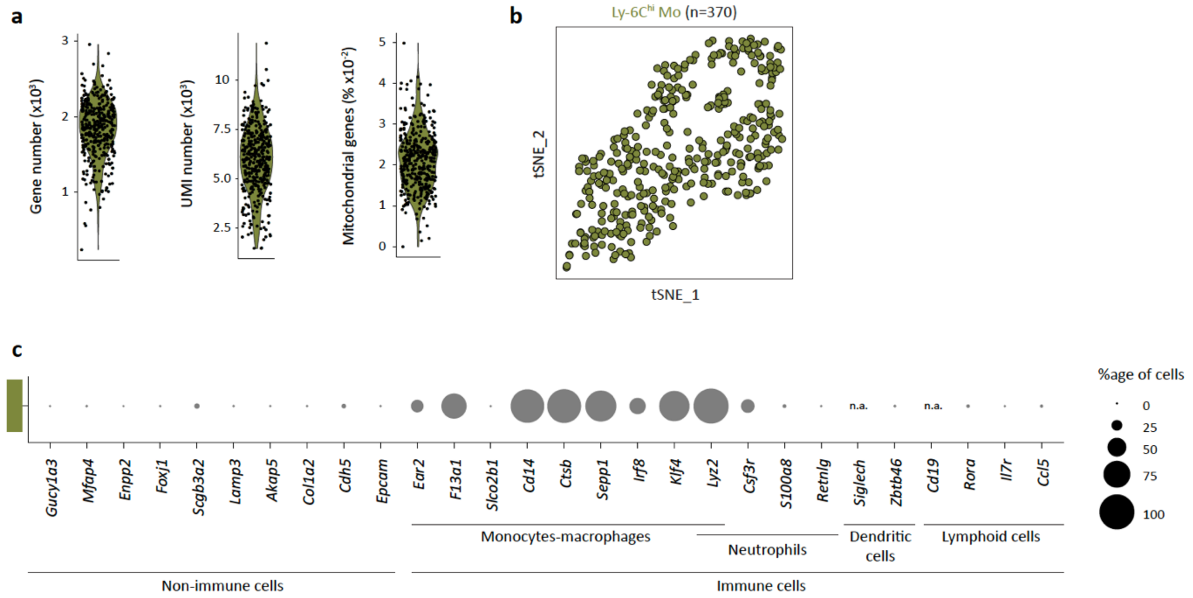

Supplementary Figure 12 scRNA-seq analysis of lung Ly-6C^{hi} classical monocytes (also see Supplementary Table 1 for gating strategies). **a** Gene numbers (left), Unique Molecular Identifiers (UMI) numbers (middle) and percentage of mitochondrial genes (right) detected in single cells after selection and filtering, presented as violin plots (height: gene numbers [left], UMI numbers [middle] and % Mitochondrial genes [right]; width: abundance of cells) and individual dots representing individual cells. **b** *t*-SNE plot depicting the single cells analyzed by scRNA-seq. **c** Dot plots showing average expression of the indicated genes and the percentage of cells expressing such genes, characteristic of the indicated cell types.

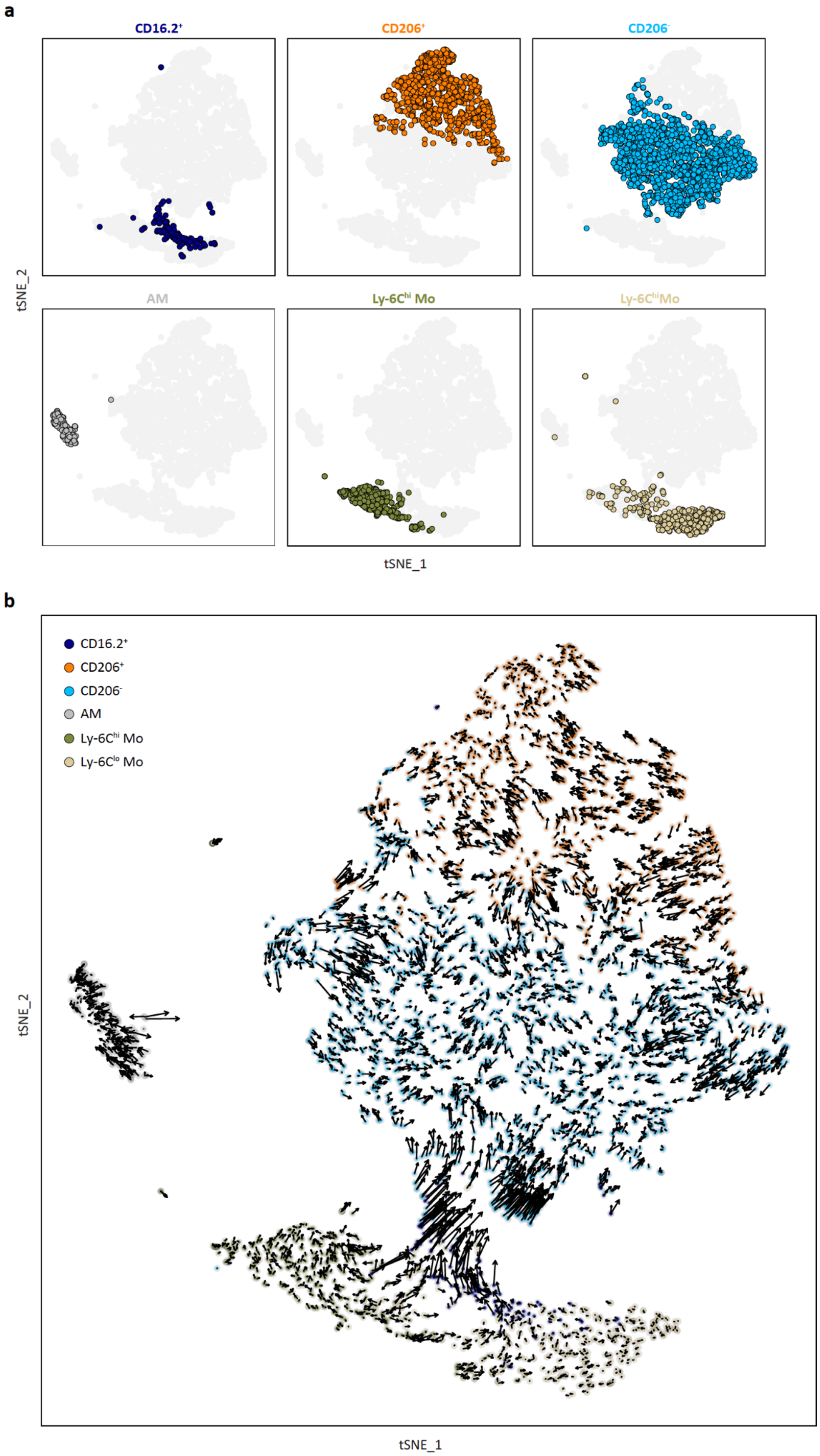
a
CD16.2+
CD206+
CD206-
AM
Ly-6Chi Mo
Ly-6ChiMo
tSNE_2
tSNE_1
b
CD16.2+
CD206+
CD206-
AM
Ly-6Chi Mo
Ly-6Clo Mo
tSNE_2
tSNE_1

Supplementary Figure 13 RNA velocity analysis of lung IM and monocyte subpopulations in steady-state C57BL/6 WT mice. **a** *t*-SNE plots depicting the merged scRNA-seq data of lung CD64-expressing cells (see Fig. 1c, encompassing AM, $CD206^-$ IM, $CD206^+$ IM, $CD64^+CD16.2^+$ monocytes), Ly-$6C^{lo}$ patrolling monocytes (see Supplementary Figure 9) and Ly-$6C^{hi}$ classical monocytes (see Supplementary Figure 12). Each individual (sub)population is highlighted separately. **b** Single cell RNA velocities, predictive of the extrapolated future states, are substantiated by arrows and embedded on the *t*-SNE plot shown in Fig. 6a.

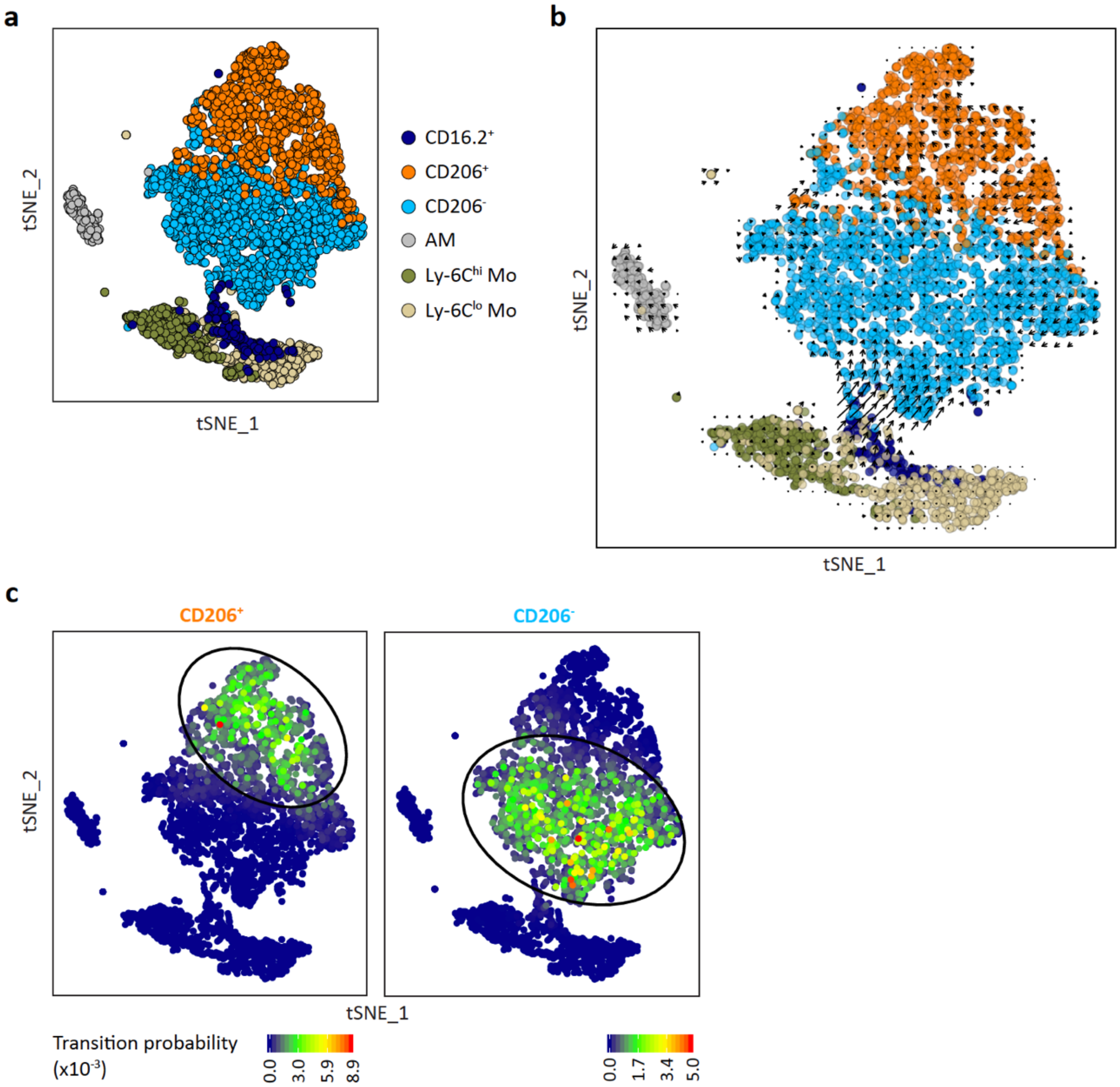

Supplementary Figure 14 RNA velocity analysis of lung IM subsets in steady-state C57BL/6 WT mice: transition probabilities. **a** *t*-SNE plot depicting the merged scRNA-seq data of lung CD64-expressing cells (see Fig. 1c), Ly-6C^{lo} patrolling monocytes (see Supplementary Fig. 9) and Ly-6C^{hi} classical monocytes (see Supplementary Fig. 12). **b** Prevalent patterns of RNA velocities are substantiated by arrows and visualized on the same *t*-SNE plot as shown in (**a**). Single cell velocities are shown in Supplementary Figure 13b. **c** Visualization of single-step transition probabilities from CD206^{+} cells (left) or CD206^{-} cells (right) to neighboring cells. Ellipses represent 95% confidence.

Supplementary Table 1 Flow cytometry phenotype of the lung mononuclear phagocyte populations investigated in this study.

(Sub)population name	Phenotype assessed by flow cytometry
IM	singlet mononuclear cell-enriched $CD45^+$ non-autofluorescent SSC^{lo} $F4/80^+$ $CD11c^-$ Ly-$6C^{lo}$ $CD64^+$
$CD16.2^+$	singlet mononuclear cell-enriched $CD45^+$ non-autofluorescent SSC^{lo} $F4/80^+$ $CD11c^-$ Ly-$6C^{lo}$ $CD64^+$ $CD16.2^+$
$CD206^+$	singlet mononuclear cell-enriched $CD45^+$ non-autofluorescent SSC^{lo} $F4/80^+$ $CD11c^-$ Ly-$6C^{lo}$ $CD64^+$ $CD206^+$
CD206-	singlet mononuclear cell-enriched $CD45^+$ non-autofluorescent SSC^{lo} $F4/80^+$ $CD11c^-$ Ly-$6C^{lo}$ $CD64^+$ $CD16.2^-$ $CD206^-$
AM	singlet mononuclear cell-enriched $CD45^+$ autofluorescent $CD11c^{hi}$
Ly-$6C^{hi}$ Mo	singlet mononuclear cell-enriched $CD45^+$ non-autofluorescent SSC^{lo} $F4/80^+$ $CD11c^-$ Ly-$6C^{hi}$ $CD64^-$
Ly-$6C^{lo}$ Mo	singlet mononuclear cell-enriched $CD45^+$ non-autofluorescent SSC^{lo} $F4/80^+$ $CD11c^-$ Ly-$6C^{lo}$ $CD64^-$
DC	singlet mononuclear cell-enriched $CD45^+$ non-autofluorescent SSC^{lo} $F4/80^-$ $CD11c^{hi}$

Supplementary Table 2 GO enrichment analysis of IM-related clusters: significantly enriched genes involved in each biological process.

Cluster	GO Term	Biological Process	Genes
1	GO:0006952	defense response	*Ccrl2 ; H2-Aa ; Mpeg1 ; Cd44 ; Actg1 ; Cxcl2 ; Capg ; Ccr2 ; Cxcl16 ; Clec4n ; Serpine1 ; Cd74 ; Ptgs2 ; Axl ; Ccl22 ; Lgals3 ; Ptafr ; Tnfaip8 ; H2-Eb1 ; Il1b ; Ly86 ; Cotl1 ; Itgax ; Cd14 ; Slamf7 ; Anxa1 ; Vim ; Nlrp3 ; Acp5 ; Tlr2 ; Il1rn ; H2-Ab1 ; Lsp1*
	GO:0050863	regulation of T cell activation	*H2-Aa ; Cd44 ; Sdc4 ; Ccr2 ; H2-DMa ; Tnfsf9 ; Cd74 ; Lgals3 ; Il1b ; Tnfsf13b ; Anxa1 ; Nlrp3 ; Cd83 ; H2-Ab1*
	GO:0019882	antigen processing and presentation	*H2-Aa ; H2-DMb2 ; H2-DMa ; Cd74 ; H2-Eb1 ; H2-M2 ; H2-DMb1 ; Ctss ; H2-Ab1*
	GO:0071222	cellular response to lipopolysaccharide	*Cxcl2 ; Cxcl16 ; Serpine1 ; Tnip3 ; Axl ; Nfkb1 ; Il1b ; Cd14 ; Vim ; Nlrp3 ; Il1rn*
2	GO:0006898	receptor-mediated endocytosis	*Folr2 ; Eps15 ; Cltc ; Cd36 ; Apoe ; Msr1 ; Ap2a2 ; Siglech ; Dab2 ; Mrc1 ; Clta ; Lrp6*
	GO:0009611	response to wounding	*Hmox1 ; P2ry12 ; Zfp36l1 ; Zfp36 ; Ccl2 ; Pf4 ; Thbd ; Jun ; Hbegf ; F13a1 ; Gas6 ; Sulf2 ; Ninj1 ; Clec10a ; Igf1*
	GO:0002690	positive regulation of leukocyte chemotaxis	*Cxcl13 ; Ccl2 ; Ccr1 ; Pla2g7 ; C3ar1 ; Lgmn ; Ccl4 ; Gas6*
	GO:0071222	cellular response to lipopolysaccharide	*Abca1 ; Il10 ; Cxcl13 ; Zfp36 ; Ccl2 ; Cd36 ; Ly96 ; Pf4 ; Ccl12 ; Mrc1 ; Arid5a*
4	GO:0007159	leukocyte cell-cell adhesion	*Itga4 ; Ptprc ; Itgal ; Rac2 ; Syk ; Stk10 ; Il1b ; Itgb2 ; Sema4d ; Spn ; Itgb7 ; Msn*
	GO:0007229	integrin-mediated signaling pathway	*Itga4 ; Fgr ; Itgal ; Syk ; Ptk2b ; Itgb2 ; Rhoa ; Tln1 ; Tyrobp ; Fcer1g ; Itgb7 ; Fyb ; Zyx*
	GO:0051495	positive regulation of cytoskeleton organization	*Pfn1 ; Arpc3 ; Arpc2 ; Cd47 ; Pam3k1 ; Stap1 ; Rps3 ; Cdc42ep3 ; Ptk2b ; Flna ; Arhgef10l ; Cdc42ep2 ; Evl ; Rhoa ; Cdkn1b ; Gda ; Arpc1b ; Arpc5 ; Hck*
	GO:0097529	myeloid leukocyte migration	*Rps19 ; Stat5b ; Cd47 ; Syk ; Il17ra ; Il1b ; Ccl6 ; Itgb2 ; Trem1 ; Trem3 ; Fcer1g ; Rpl13a ; Gpr35*

Supplementary Table 3 List of reagents and antibodies used in this study.

Reagent	Source	Cat. number
**Antibodies and secondary reagents**		
Mouse anti-mouse CD45.2, V500, clone 104	BD Pharmingen	562129
Mouse anti-mouse CD45.2, PE-Cy7, clone 104	BD Pharmingen	560696
Mouse anti-mouse CD45.2, APC, clone 104	BD Pharmingen	558702
Mouse anti-mouse CD45.1, APC, clone A20	BD Pharmingen	558701
Rat anti-mouse F4/80, PE, clone BM8	Sony Biotechnology	1215550
Rat anti-mouse F4/80, BV605, clone BM8	Biolegend	123133
Hamster anti-mouse CD11c, APC-Cy7, clone HL3	BD Pharmingen	561241
Rat anti-mouse Ly-6C, PerCP-Cy5.5, clone AL-21	BD Pharmingen	560525
Mouse anti-mouse CD64, BV421, clone X54-5/7.1	Sony Biotechnology	1296545
Mouse anti-mouse CD64 a and b, PE, clone XB54-5/7.1.1	BD Pharmingen	558455
Armenian Hamster anti-mouse CD16.2, AF647, clone 9E9	Biolegend	149526
Armenian Hamster anti-mouse CD16.2, AF488, clone 9E9	Biolegend	149523
Rat anti-mouse CD206, AF488, clone C068C2	Biolegend	141710
Rat anti-mouse CD206, AF647, clone C068C2	Biolegend	141712
Rat anti-mouse CD206, PE-Cy7, clone C068C2	Biolegend	141719
Mouse anti-mouse I-Ab, AF647, clone AF6-120.1	Biolegend	116412
Mouse anti-mouse I-Ab, AF488, clone AF6-120.1	Biolegend	116410
Mouse anti-mouse I-Ab, PE-Cy7, clone AF6-120.1	Biolegend	116420
Rat anti-mouse ACE, AF647, clone 230214	R&D Systems	FAB15131R
Rabbit anti-mouse/human FOLR2, purified, polyclonal	Biorbyt	orb35326
Rat anti-mouse CD107a (LAMP-1), PE, clone 1D4B	BD Pharmingen	558661
Rat anti-mouse CD11b, PE-Cy7, clone M1/70	BD Pharmingen	552850
Rat anti-mouse CD11b, V450, clone M1/70	BD Pharmingen	560455
Rat anti-mouse CD115, biotin, clone AFS98	Thermo Fisher Scientific	13-1152-82
Rat anti-mouse CD115, APC, clone AFS98	ThermoFisherScientific	17-1152-82
Goat anti-mouse MerTK, biotin, polyclonal	R&D Systems	BAF591
Rat anti-mouse CD68, PE-Cy7, clone FA-11	Biolegend	137015
Rat anti-mouse CD68, purified, clone FA-11	Biorad	MCA1957GA
Rat anti-mouse Ly-6G, PE-Cy7, clone 1A8	BD Pharmingen	560601
Rat anti-mouse CD19, APC-Cy7, clone 1D3	BD Pharmingen	557655
Rat anti-mouse Siglec-F, PE, clone E50-2440	BD Pharmingen	552126
Rat anti-mouse Ki-67, PE, clone 16A8	Biolgend	652403
Rabbit anti-GFP, AF488, polyclonal	Thermo Fisher Scientific	A21311
Rat anti-mouse CD31, AF647, clone MEC13.3	Biolegend	102515
Rat anti-mouse Lyve1, eFluor660, clone ALY7	Thermo Fisher Scientific	5016437
Mouse anti-mouse TUBB3, AF647, clone AA10	Biolegend	657406
Rat anti-mouse/human CD11b microbeads	Miltenyi Biotec	130-049-601
Donkey anti-rabbit IgG, AF647, clone Poly4064	Biolegend	406414
Goat anti-rat IgG, AF568, polyclonal	Thermo Fisher Scientific	A11077
Streptavidin, PE-Cy7	BD Pharmingen	557598
**Isotypes**		
Rat IgG2a κ, biotin, clone R35-95	BD Pharmingen	553928
Rat IgG2a κ, PE, clone eBR2a	ThermoFisherScientific	12-4321-81
Rat IgG2b κ, PE-Cy7, clone RTK4530	Biolegend	400617
Rat IgG2a , PE-Cy7, clone RTK 2758	Biolegend	400521
Armenian Hamster IgG, AF647, clone HTK888	Biolegend	400924
Goat IgG, biotin, polyclonal	R&D Systems	BAF108
**Media, chemicals and other reagents**		
Calcium- and magnesium-free PBS	Gibco	14190-094
HBSS with Phenol Red	Lonza	BE10-508F

RPMI with L-glutamine	Lonza	BE12-702F
Fetal Bovine Serum	Gibco	10270-098
Goat serum	Sigma	G9023
Collagenase A	Roche	10103586001
DNase I	Roche	11284932001
EDTA	Merck Millipore	1084181000
Bovine Serum Albumin	Sigma	A7906
UltraPure Bovine Serum Albumin	Thermo Fisher Scientific	AM2616
Sodium azide	Acros Organics	190385000
Mouse Fc block (purified rat anti-mouse CD16/CD32)	BD Pharmingen	553142
Brilliant Stain Buffer	BD Pharmingen	563794
Fixation Buffer	Biolegend	420801
Foxp3/Transcription Factor Staining Buffer Set	Thermo Fisher Scientific	00-5523-00
CCF4-AM	Thermo Fisher Scientific	K1028
Probenecid	Thermo Fisher Scientific	P36400
Ammonium chlorure	VWR	21236267
Potassium bicarbonate	Sigma	60338
Percoll	GE Healthcare	17089101
Hemacolor Solution 1 – fixing solution	Merck	111955
Hemacolor Solution 2 – colour reagent red	Merck	111956
Hemacolor Solution 3 – colour reagent blue	Merck	111957
2-mercaptoethanol	Gibco	31350-010
MEM NEAA	Gibco	11140-035
Sodium pyruvate	GE Healthcare	SH30239.01
Penicillin/streptomycin	Gibco	15070-063
Proteome profiler mouse XL cytokine array	R&D Systems	ARY028
Q Path Freeze gel	VWR	07111247
Triton X-100	Sigma	93443
DAPI	Biolegend	422801
Lipopolysaccharides from *E.coli* O55:B5	Sigma	L4524
pHrodo™ Green *E.coli* BioParticules™	Thermo Fisher Scientific	P35366
CpG-DNA, Mouse	Hycult Biotech	HC4033
PAM3CSK4	InvivoGen	tlr-pms
Tamoxifen	Sigma	T5648
Corn oil	Sigma	C8267
DynaBeads MyOne Silane	Thermo Fisher Scientific	37002D
SPRIselect Reagent	Beckman Coulter	B23317
Agilent High Sensitivity DNA Kit	Agilent	5067-4626

Supplementary References

1. Cohen, M. *et al.* Lung Single-Cell Signaling Interaction Map Reveals Basophil Role in Macrophage Imprinting. *Cell* **175**, 1031-1044 e18 (2018).
2. Scott, C. L. *et al.* The Transcription Factor ZEB2 Is Required to Maintain the Tissue-Specific Identities of Macrophages. *Immunity* **49**, 312-325 e5 (2018).
3. Schneider, C. *et al.* Induction of the nuclear receptor PPAR-gamma by the cytokine GM-CSF is critical for the differentiation of fetal monocytes into alveolar macrophages. *Nat Immunol* **15**, 1026–37 (2014).
4. Chakarov, S. *et al.* Two distinct interstitial macrophage populations coexist across tissues in specific subtissular niches. *Science* **363**, (2019).
5. Gibbings, S. L. *et al.* Three Unique Interstitial Macrophages in the Murine Lung at Steady State. *Am J Respir Cell Mol Biol* **57**, 66–76 (2017).
