# Supplementary material for: Non-classical tissue monocytes and two functionally distinct populations of interstitial macrophages populate the mouse lung
Source: Nat Commun. 2019 Sep 3;10:3964. doi: 10.1038/s41467-019-11843-0 (PMC6722135; doi:10.1038/s41467-019-11843-0)
Supplement: Supplementary file 2 — Reporting Summary [file 41467_2019_11843_MOESM2_ESM.pdf]

nature research

Corresponding author(s): Thomas Marichal

Last updated by author(s): 2019/08/01

Reporting Summary

Nature Research wishes to improve the reproducibility of the work that we publish. This form provides structure for consistency and transparency in reporting. For further information on Nature Research policies, see Authors & Referees and the Editorial Policy Checklist.

Statistics

For all statistical analyses, confirm that the following items are present in the figure legend, table legend, main text, or Methods section.

n/a	Confirmed	
☐	☒	The exact sample size (n) for each experimental group/condition, given as a discrete number and unit of measurement
☐	☒	A statement on whether measurements were taken from distinct samples or whether the same sample was measured repeatedly
☐	☒	The statistical test(s) used AND whether they are one- or two-sided<br>*Only common tests should be described solely by name; describe more complex techniques in the Methods section.*
☒	☐	A description of all covariates tested
☐	☒	A description of any assumptions or corrections, such as tests of normality and adjustment for multiple comparisons
☐	☒	A full description of the statistical parameters including central tendency (e.g. means) or other basic estimates (e.g. regression coefficient) AND variation (e.g. standard deviation) or associated estimates of uncertainty (e.g. confidence intervals)
☐	☒	For null hypothesis testing, the test statistic (e.g. F, t, r) with confidence intervals, effect sizes, degrees of freedom and P value noted<br>*Give P values as exact values whenever suitable.*
☒	☐	For Bayesian analysis, information on the choice of priors and Markov chain Monte Carlo settings
☒	☐	For hierarchical and complex designs, identification of the appropriate level for tests and full reporting of outcomes
☒	☐	Estimates of effect sizes (e.g. Cohen's d, Pearson's r), indicating how they were calculated

Our web collection on statistics for biologists contains articles on many of the points above.

Software and code

Policy information about availability of computer code

Data collection	FACSDIVA software (version 8.0.1, BD Biosciences) was used for flow cytometry data collection. ZEN software (version 2.3, Zeiss) was used for confocal images acquisition. ImageQuant LAS 4000 was used for chemiluminescence acquisition for proteome profiler experiments. Cell Ranger software (version 1.2.0, 10x Genomics) was used to demultiplex Illumina BCL files to FASTQ files (cellranger mkfastq), to perform alignment (to mouse GRCm38/mm10 genome), filtering, UMI counting and to produce gene – barcode matrices (cellranger count).
Data analysis	FlowJo software (version 10, Tree Star Inc.) was used for flow cytometry data analysis. ZEN software (version 2.3, Zeiss) was used for confocal images analysis. ImageJ sofwtare (version 1.51j8) was used to analyze the images acquired during proteome profiler experiments. (Single cell) RNA-seq analysis used R bioconductor (version 3.4.2.), and the R package Seurat (version 2.1.0) or DESeq2. Gene ontology analyses were performed using the Gene Ontology Consortium website (http://geneontology.org/) referring to the GO Ontology database released on 2018/12/01. Statistical analysis were performed using Prism software (version 7, Graphpad), R bioconductor (version 3.4.2.) or SAS (version 9.3).

For manuscripts utilizing custom algorithms or software that are central to the research but not yet described in published literature, software must be made available to editors/reviewers. We strongly encourage code deposition in a community repository (e.g. GitHub). See the Nature Research guidelines for submitting code & software for further information.

Data

Policy information about availability of data

All manuscripts must include a data availability statement. This statement should provide the following information, where applicable:

- Accession codes, unique identifiers, or web links for publicly available datasets
- A list of figures that have associated raw data
- A description of any restrictions on data availability

The scRNA-seq data provided in this manuscript have been deposited in the ArrayExpress database at EMBL-EBI (https://www.ebi.ac.uk/arrayexpress/experiments/

E-MTAB-7678) under accession number E-MTAB-7678.

Field-specific reporting

Please select the one below that is the best fit for your research. If you are not sure, read the appropriate sections before making your selection.

☒ Life sciences ☐ Behavioural & social sciences ☐ Ecological, evolutionary & environmental sciences

For a reference copy of the document with all sections, see nature.com/documents/nr-reporting-summary-flat.pdf

Life sciences study design

All studies must disclose on these points even when the disclosure is negative.

Sample size	We assume that variance will be equal between experimental groups. We estimate that a difference will be biologically significant between two groups if a variation of at least 50% is observed between the mean of the two groups. If we want to compare two means of 100.000 and 200.000 with a standard deviation of 50.000 (example from the number of classical monocytes in the lung of Ccr2-/- and WT mice) , the results of power analysis for expecting effects show that for a power of 0.9 (and α<0.05) in a t student test, the required sample size would be 3-5 animals.
Data exclusions	No data were excluded for the analyses
Replication	For each experiment, each experimental group was composed of 2-5 mice constituting biological replicates. All experiments have been repeated at least two times. All attempts at replication were successful.
Randomization	Age- and sex-matched C57Bl/6J mice were randomly allocated into experimental groups.
Blinding	Blinding was not possible in these experiments since mice were dispatched in the different conditions by the manipulator who processed and analyzed the samples. Of note, for some experiments (involving Cx3cr1GFP/GFP, Cx3cr1CreERT2.Rosa26-LSL-YFP, IL-10-β-lactamase reporter (ITIB) mice, or the injection of fluorescent dies), control mice had to be clearly identified since some flow cytometry/confocal microscopy controls needed to be performed only on cells/tissues from these mice.

Reporting for specific materials, systems and methods

We require information from authors about some types of materials, experimental systems and methods used in many studies. Here, indicate whether each material, system or method listed is relevant to your study. If you are not sure if a list item applies to your research, read the appropriate section before selecting a response.

Materials & experimental systems

n/a	Involved in the study	
☐	☒	Antibodies
☒	☐	Eukaryotic cell lines
☒	☐	Palaeontology
☐	☒	Animals and other organisms
☒	☐	Human research participants
☒	☐	Clinical data

Methods

n/a	Involved in the study	
☒	☐	ChIP-seq
☐	☒	Flow cytometry
☒	☐	MRI-based neuroimaging

Antibodies

Antibodies used

Mouse anti-mouse CD45.2, V500, clone 104 BD Pharmingen 562129
Mouse anti-mouse CD45.2, PE-Cy7, clone 104 BD Pharmingen 560696
Mouse anti-mouse CD45.2, APC, clone 104 BD Pharmingen 558702
Mouse anti-mouse CD45.1, APC, clone A20 BD Pharmingen 558701
Rat anti-mouse F4/80, PE, clone BM8 Sony Biotechnology 1215550
Rat anti-mouse F4/80, BV605, clone BM8 Biolegend 123133
Hamster anti-mouse CD11c, APC-Cy7, clone HL3 BD Pharmingen 561241
Rat anti-mouse Ly-6C, PerCP-Cy5.5, clone AL-21 BD Pharmingen 560525
Mouse anti-mouse CD64, BV421, clone X54-5/7.1 Sony Biotechnology 1296545
Mouse anti-mouse CD64 a and b, PE, clone XB54-5/7.1.1 BD Pharmingen 558455
Armenian Hamster anti-mouse CD16.2, AF647, clone 9E9 Biolegend 149526
Armenian Hamster anti-mouse CD16.2, AF488, clone 9E9 Biolegend 149523
Rat anti-mouse CD206, AF488, clone C068C2 Biolegend 141710
Rat anti-mouse CD206, AF647, clone C068C2 Biolegend 141712
Rat anti-mouse CD206, PE-Cy7, clone C068C2 Biolegend 141719
Mouse anti-mouse I-Ab, AF647, clone AF6-120.1 Biolegend 116412
Mouse anti-mouse I-Ab, AF488, clone AF6-120.1 Biolegend 116410

Mouse anti-mouse I-Ab, PE-Cy7, clone AF6-120.1 Biolegend 116420
Rat anti-mouse ACE, AF647, clone 230214 R&D Systems FAB15131R
Rabbit anti-mouse/human FOLR2, purified, polyclonal Biorbyt orb35326
Rat anti-mouse CD11b, PE-Cy7, clone M1/70 BD Pharmingen 552850
Rat anti-mouse CD115, biotin, clone AFS98 Thermo Fisher Scientific 13-1152-82
Goat anti-mouse MerTK, biotin, polyclonal R&D Systems BAF591
Rat anti-mouse CD68, PE-Cy7, clone FA-11 Biolegend 137015
Rat anti-mouse CD68, purified, clone FA-11 Biorad MCA1957GA
Rat anti-mouse Ly-6G, PE-Cy7, clone 1A8 BD Pharmingen 560601
Rat anti-mouse CD19, APC-Cy7, clone 1D3 BD Pharmingen 557655
Rat anti-mouse Siglec-F, PE, clone E50-2440 BD Pharmingen 552126
Rat anti-mouse Ki-67, PE, clone 16A8 Biolgend 652403
Rabbit anti-GFP, AF488, polyclonal Thermo Fisher Scientific A21311
Rat anti-mouse CD31, AF647, clone MEC13.3 Biolegend 102515
Rat anti-mouse Lyve1, eFluor660, clone ALY7 Thermo Fisher Scientific 5016437
Mouse anti-mouse TUBB3, AF647, clone AA10 Biolegend 657406
Rat anti-mouse/human CD11b microbeads Miltenyi Biotec 130-049-601
Donkey anti-rabbit IgG, AF647, clone Poly4064 Biolegend 406414
Goat anti-rat IgG, AF568, polyclonal Thermo Fisher Scientific A11077
Streptavidin, PE-Cy7 BD Pharmingen 557598
Rat IgG2a κ, biotin, clone R35-95 BD Pharmingen 553928
Rat IgG2a κ, PE, clone eBR2a ThermoFisherScientific 12-4321-81
Rat IgG2b κ, PE-Cy7, clone RTK4530 Biolegend 400617
Rat IgG2a , PE-Cy7, clone RTK 2758 Biolegend 400521
Armenian Hamster IgG, AF647, clone HTK888 Biolegend 400924
Goat IgG, biotin, polyclonal R&D Systems BAF108

Validation

All the antibodies are from commercial sources and purchased from Biolegend, BD Pharmingen, Thermo Fisher Scientific, R&D Systems, Biorad, Sony Biotechnology, Biorbyt and Miltenyi Biotec. The data sheets were provided by the manufacturer where the validation of antibodies was confirmed.

Animals and other organisms

Policy information about studies involving animals; ARRIVE guidelines recommended for reporting animal research

Laboratory animals

Females C57BL/6J WT (CD45.2), Nr4a1-/- (CD45.2), CD45.1, CD45.1/2, Il10-/-, Cx3cr1GFP/GFP, IL-10-β-lactamase reporter (ITIB) mice, females and males Ccr2-/- and Cx3cr1CreERT2.Rosa26-LSL-YFP mice were used at 7-11 weeks of age, unless otherwise indicated.

Wild animals

This study didn't involve wild animals.

Field-collected samples

That study didn't involve samples collected from the field.

Ethics oversight

All animals and experimental procedures were reviewed and approved by the Institutional Animal Care and Use Committee of the University of Liège, except fate-mapping experiments involving Cx3cr1CreERT2.Rosa26-LSL-YFP mice which were approved by the Animal Care and Animal Experiments Committee of the Medical Faculty of the KU Leuven

Note that full information on the approval of the study protocol must also be provided in the manuscript.

Flow Cytometry

Plots

Confirm that:

☒ The axis labels state the marker and fluorochrome used (e.g. CD4-FITC).

☒ The axis scales are clearly visible. Include numbers along axes only for bottom left plot of group (a 'group' is an analysis of identical markers).

☒ All plots are contour plots with outliers or pseudocolor plots.

☒ A numerical value for number of cells or percentage (with statistics) is provided.

Methodology

Sample preparation

To obtain single-lung-cell suspensions, lungs were extensively perfused with 3 ml of HBSS (Lonza) through the right ventricle, cut into small pieces with razor blades, and digested for 1 h at 37°C in HBSS containing 5% v/v of FBS (Gibco), 1 mg.ml-1 collagenase A (Roche,) and 0.05 mg.ml-1 DNase I (Roche). The suspension was then enriched in mononuclear cells by harvesting cells from the 1.080:1.038 g.ml-1 interface using a density gradient (Percoll from GE Healthcare). Staining reactions were performed at 4°C with 2% v/v of Fc block (BD Pharmingen) to reduce non-specific binding. White blood cells were analyzed after red blood cell lysis.

Instrument	Cell phenotyping was performed on a FACSLSRFortessa (BD Biosciences) and cell sorting was performed on a FACSAriaIII (BD Biosciences)
Software	FACSDIVA software v8.0.1 (BD Biosciences) and FlowJo software v10 (Tree Star Inc.) were used for flow cytometry data collection and analysis respectively.
Cell population abundance	We sorted a minimum of 13.000 cells for one condition. The purity of sorted samples was above 95% for every samples.
Gating strategy	Lung tissue CD64-expressing cells were identified as singlet mononuclear cell-enriched CD45+ non-autofluorescent SSCloF4/80 +CD11c-Ly-6CloCD64+, as shown in Figure 1a. The gating strategy of lung tissue CD64-expressing cells subsets (i.e. CD206+, CD206- and CD16.2+), alveolar macrophages, dendritic cells, classical and patrolling monocytes are explained in the Supplementary Table 1. In the blood, B lymphocytes, Ly-6Clo monocytes, Ly-6Chi monocytes and neutrophils, were defined as CD45+CD11b-Ly-6G-CD19+, CD45+CD11b+Ly-6G-CD115+Ly-6C-, CD45+CD11b+Ly-6G-CD115+Ly-6C+ and CD45+CD11b+ Ly-6G+ cells, respectively. For adoptive transfer experiments, classical and patrolling monocytes were FACS-sorted as CD45+CD11b +CD115+Ly-6C+ and CD45+CD11b+CD115+Ly-6C- cells, respectively.

☒ Tick this box to confirm that a figure exemplifying the gating strategy is provided in the Supplementary Information.
